# Development of a universal and simplified ddRAD library preparation approach for SNP discovery and genotyping in angiosperm plants

**DOI:** 10.1186/s13007-016-0139-1

**Published:** 2016-08-04

**Authors:** Guo-Qian Yang, Yun-Mei Chen, Jin-Peng Wang, Cen Guo, Lei Zhao, Xiao-Yan Wang, Ying Guo, Li Li, De-Zhu Li, Zhen-Hua Guo

**Affiliations:** 1 Germplasm Bank of Wild Species, Kunming Institute of Botany, Chinese Academy of Sciences, Kunming, 650201 China; 2 Kunming College of Life Sciences, University of Chinese Academy of Sciences, Kunming, 650201 China; 3 Key Laboratory of Experimental Marine Biology, Institute of Oceanology, Chinese Academy of Sciences, Qingdao, 266071 China

**Keywords:** RAD-seq, ddRAD, *Mi*ddRAD, Genotype-by-sequencing, Next-generation sequencing

## Abstract

**Background:**

The double digest restriction-site associated DNA sequencing technology (ddRAD-seq) is a reduced representation sequencing technology by sampling genome-wide enzyme loci developed on the basis of next-generation sequencing. ddRAD-seq has been widely applied to SNP marker development and genotyping on animals, especially on marine animals as the original ddRAD protocol is mainly built and trained based on animal data. However, wide application of ddRAD-seq technology in plant species has not been achieved so far. Here, we aim to develop an optimized ddRAD library preparation protocol be accessible to most angiosperm plant species without much startup pre-experiment and costs.

**Results:**

We first tested several combinations of enzymes by in silico analysis of 23 plant species covering 17 families of angiosperm and 1 family of bryophyta and found *AvaII* + *MspI* enzyme pair produced consistently higher number of fragments in a broad range of plant species. Then we removed two purifying and one quantifying steps of the original protocol, replaced expensive consumables and apparatuses by conventional experimental apparatuses. Besides, we shortened P1 adapter from 37 to 25 bp and designed a new barcode-adapter system containing 20 pairs of barcodes of varying length. This is an optimized ddRAD strategy for angiosperm plants that is economical, time-saving and requires little technical expertise or investment in laboratory equipment. We refer to this simplified protocol as *Mi*ddRAD and we demonstrated the utility and flexibility of our approach by resolving phylogenetic relationships of two genera of woody bamboos (*Dendrocalamus* and *Phyllostachys*). Overall our results provide empirical evidence for using this method on different model and non-model plants to produce consistent data.

**Conclusions:**

As *Mi*ddRAD adopts an enzyme pair that works for a broad range of angiosperm plants, simplifies library constructing procedure and requires less DNA input, it will greatly facilitate designing a ddRAD project. Our optimization of this method may make ddRAD be widely used in fields of plant population genetics, phylogenetics, phylogeography and molecular breeding.

**Electronic supplementary material:**

The online version of this article (doi:10.1186/s13007-016-0139-1) contains supplementary material, which is available to authorized users.

## Background

Restriction-site associated DNA sequencing technology (RAD-seq) is a reduced representation sequencing technology by sampling genome-wide single enzyme loci developed on the basis of next-generation sequencing [1, 2]. The technology breaks genome into a certain size of DNA fragments by employing a restriction endonuclease (usually a low-frequency cutter) combined with the ultrasonic shearing method, then the fragmented DNA is enriched for constructing a sequencing library so that sequences beside the cleavage site can be acquired for high-throughput sequencing [3]. Because RAD-tags are DNA fragments beside a specific restriction site from the whole genome, so they can generally reflect the sequence characteristics of the entire genome. It is now possible to obtain hundreds to thousands of single nucleotide polymorphism (SNP) markers within a species or between closely related species through RAD-seq. Until now RAD-seq has been successfully applied to SNP marker development, high-density genetic map construction, QTL mapping, population genetics and phylogenetic research on eggplants, chickpeas, sesames, soybeans, cucurbit bottle gourds, bamboos, beetles, and other organisms [4–13]. But on one aspect experimental procedure of this technology is much complex and it requires a Covaris ultrasonicator and some other specialized instruments, so personnels under professional training are usually required to master the technique; on the other hand, random physical shearing methods implemented in the library construction process will result in losing lots of DNA, thereby leading to out control of the final tag number [3, 14]. So several laboratories have improved and simplified the traditional RAD-seq method, from which a variety of low cost, high throughput reduced representation sequencing methods are available. At present, reduced representation sequencing methods developed from the RAD-seq mainly includes GBS series techniques and RAD series techniques [14]. GBS and RAD-seq techniques share several basic steps while differ only in the order or details of enzyme digestion, adapter ligation, barcoding and size selection. Each alternative RAD method has both advantages and drawbacks. RAD series control number of the tags by both choosing the enzyme and size selection while GBS series techniques or close derivatives control the number of tags only by selecting different enzymes (though some GBS users may also add a size selection step in their modified GBS protocol [15], the original intention of GBS is to reduce library preparation workflow without size selection). GBS series techniques include single and double enzyme GBS [16, 17], both of which employ simple library constructing processes, but they can only enrich small fragments less than ~350 bp [18]. It’s easy to sequence through the short fragment with pair-end sequencing mode as the sequencing length is gradually becoming longer, which will result in a waste of data and the potential to discover more SNPs. Furthermore, fragments of various lengths will increase the potential for amplification bias [19, 20] and cause a decline in the data quantity and data quality. RAD series mainly includes 2b-RAD [21], ddRAD [22] and ezRAD [23]. 2b-RAD adopts a kind of type II restriction endonuclease to digest the genome, producing only ~33 bp fragments, which lack of biases due to fragment size selection but may restrict the potential for discovering more SNPs. ezRAD is the only protocol that relies on illumina authoritative kits to construct the library with customer support but the cost is still not as low as the author claimed [24]. ddRAD can tune fragments number by employing two different enzymes and size selection, and the process of constructing a library is quite simple while genomic DNA it requires is of the highest quality in all the RAD methods [24].

All RAD protocols have been proved to be powerful tools for SNP discovery and genotyping of model and non-model species. However, startup of them all usually involves pre-experiment of (1) testing candidate endonuclease that could produce a suitable RAD or GBS library [25], and (2) purchasing some relatively expensive consumables and apparatus (e.g. Agilent 2100 Bioanalyzer). This requires a significant initial investment for labs focused on traditional genotyping methods (e.g. SSR genotyping). Besides, many labs (e.g. phylogenetic bio-labs) are probably focusing on different model or non-model plant species, once a pair of enzymes selected and adapters purchased for one target species, they have to consider if these consumables could be applied to another species efficiently to be studied even some commonly used enzyme pairs could produce hundreds to thousands of markers across wide-range species. Enzyme pairs simulation of the original ddRAD protocol is mainly based on animal genomes and it is hard for us to know if performance of the enzyme pairs is good as well in plant species as Santiago et al. found that a given restriction enzyme may have strikingly variable recognition-sequence frequencies among broad eukaryotic taxonomic groups, and only phylogenetic related species could produce similar recognition-sequence frequencies [26]. In another study, Burford et al. found some enzyme pairs work more consistently than others across a wide range of taxonomic groups after optimizing ddRAD protocol and testing several restriction enzyme pairs for five genera of insects and fish [27]. Here, we sought to test the universality of several commonly used enzyme pairs across most angiosperm plants, simplify the ddRAD protocol and reduce the overall costs. Our protocol is generally according to the protocol described by Peterson et al. [22], but with some modifications as we first tested several combinations of enzymes by in silico analysis of 23 plant species covering 17 families of angiosperm (16 orders, two classes) and one family of bryophyta (one orders, one class) and found *AvaII* + *MspI* enzyme pair produced consistently higher number of fragments in a broad range of plant species. Furthermore, we removed two purifying and one quantifying steps, shortened the adapters and replaced expensive instruments by conventional experimental apparatuses which make it possible to do ddRAD sequencing with no additional investment beyond the cost of library preparation and sequencing itself.

To assess the performance of this approach, we got empirical results from the model species *Oryza sativa* L. japonica and *Zea mays* L. We also explored repeatability by testing the effectiveness of the method in non-model species *Phyllostachys edulis* and *Alloteropsis semialata* (R. Br.) Hitchc. Finally, we managed to reconstruct phylogenetic relationships of two woody bamboos genera, *Dendrocalamus* and *Phyllostachys* with data generated by the protocol. This generalized approach, using the fixed enzyme pair and standard library preparation protocol, will allow researchers to apply ddRAD-seq technology to a wide array of plants and research questions. We expect that this optimized protocol could be efficiently implemented in any small or middle-sized laboratory with few people and limited funds.

## Methods

### Plant material and DNA samples

In this project, we used *Oryza sativa* L. spp. japonica and *Z. mays* L. to estimate the robustness of our Protocol B. Besides, a total of six species of Poaceae including four temperate woody bamboo species (*Chimonocalamus pallens, Phyllostachys edulis, Phyllostachys rubicunda* T. H. Wen and *Phyllostachys vivax* McClure), one tropical woody bamboo species (*Dendrocalamus latiflorus*) and one grass species *Alloteropsis semialata* (R. Br.) Hitchc. were used in our protocols as well. Leaves of temperate woody bamboos were mostly collected from plants grown in Kunming Botanical Garden (N25°07′04.9″, E102°44′15.2″) and leaves of tropical woody bamboos*, O. sativa*, *Z. mays* and *A. semialata* were collected from plants grown in our greenhouses. All necessary permits were obtained before collecting the material. Fresh leaves of all species were obtained and then dried rapidly in silica gel. The DNA was extracted with a modified CTAB method [28].

### Choosing restriction enzymes and adapter design

At first, we selected six kinds of enzyme pairs that could recognize restriction sites of different lengths including eight bases + six bases (*SbfI* + *EcoRI*), eight bases + four bases (*SbfI* + *MluCI*), six bases + four bases (*EcoRI* + *MspI*, *PstI* + *MspI*), 4.5 bases + four bases (*AvaII* + *MspI*), four bases + four bases (*NlaIII* + *MluCI*), of which *EcoRI* + *MspI* was adopted by the original ddRAD protocol and *PstI* + *MspI* was used by the two-enzyme of GBS protocol [17, 22]. Restriction enzymes included in this study are listed in Additional file 2: Table S2. Then we in silico digested genome sequences of 23 plant species covering 17 families of angiosperm (16 orders, two classes) and one family of bryophyte (one orders, one class) of different genome size with RestrictionDigest [29]. For each enzyme pair, we recorded the total number of fragments and the number of fragments between 400–700 bp that could produce in each species. The species adopted for analysis are listed in Additional file 2: Table S1. Genome scaffolds of these species were downloaded from Plantgdb [31]. Then the distribution of DNA fragments was screened by agarose gel electrophoresis after digestion of genomic DNA of some species.

Chemosynthetic oligonucleotides of P1 and P2 adapters will account for almost half of the cost due to the need for high-performance liquid chromatography (HPLC) purification and 5′-end phosphorylation. In our protocols, original P1 adapters are shortened from 37 to 25 bp (barcode length is assumed to be 5 bp) to reduce the cost of the synthesizing DNA oligos. Besides, a different barcode-adapter system containing 20 pairs of barcodes varying in length was devised, which can be used with integer times (20 * n), rather than the original 48 kinds of barcodes with equal length (see Additional file 1). This will not only increase the flexibility of barcodes for projects with diverse samples but also improve the quality of bases near the restriction site.

### Protocols of *Mi*ddRAD for next-generation sequencing

**Fig. 1:**
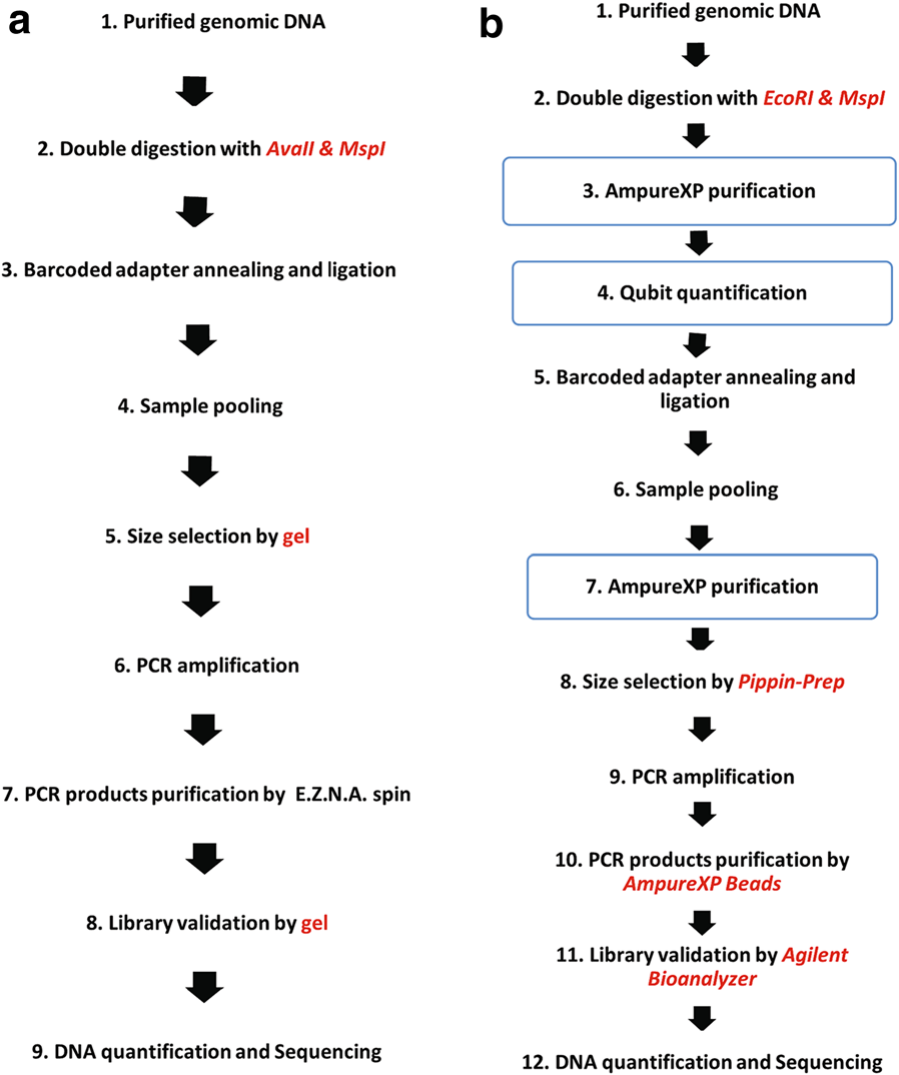
Library preparation flowchart of *Mi*ddRAD protocol B and the original ddRAD protocol. **a** *Mi*ddRAD protocol B. **b** The original ddRAD library constructing flowchart, this procedure is adopted by Peterson et al. Protocol B contains nine steps while the original ddRAD protocol contains 12 steps. Only size-selected fragments are as templates for PCR amplification in protocol B

We initially provided two protocols for constructing a library. Protocol A differs from protocol B only in when to select target fragments. In protocol A, selecting fragments was placed in the last step, i.e. products of all adapter-ligated restriction fragments were as templates of the PCR reaction; however in protocol B, only selected adapter-ligated restriction fragments were as templates for PCR amplification. Two non-model species *D. latiflorus* and *C. pallens* were used to construct libraries with protocol A while the model species *O. sativa* and *Z. mays* were used to construct libraries with protocol B (as data produced from protocol A contains too many adapters, we did not continue to verify this protocol in model plants). Figure 1 provides a flowchart that outlines all stages of protocol B and the original ddRAD. Protocol A and protocol B were detailed in Additional file 1. The protocol A flowchart was presented in Additional file 2: Figure S1. Sequencing of protocol A was performed on the Illumina HiSeq 2000 System (San Diego, CA, USA) using the pair read, 100 nucleotide configuration at Kunming Institute of Zoology, CAS while Sequencing of Protocol B was performed on the Illumina HiSeq X Ten System (San Diego, CA, USA) using the pair read, 150 nucleotide configuration at Cloud Health Genomics Ltd. To test the universality of *Mi*ddRAD and the restriction enzymes on more plant species, we constructed libraries for *P. edulis* and *A. semialata* with the same enzyme pairs. Libraries were constructed strictly according to Protocol B and were sent to Cloud Health Genomics Ltd. for sequencing using Illumina HiSeq X Ten (San Diego, CA, USA) with PE150 bp sequencing mode.

Then we adopted protocol B to construct libraries for three bamboo species (contains two *D. latiflorus* individuals, one *P. rubicunda* individual and one *P. vivax* individual) to explore the applicability of *Mi*ddRADseq-derived genotypes/markers in resolving phylogenetic problems. The library constructing process is according to Protocol B and fragments selected were set to 600–700 bp. Reagents and enzymes used were mainly purchased from New England Biolabs Inc. (R0153S, R0106S), Vazyme Biotech Co., Ltd. (C301-01) and SunShineBio Co., Ltd. (SN124). Libraries were then sequenced in Cloud Health Genomics Ltd. Sequencing platform was Illumina HiSeq X Ten (San Diego, CA, USA) with sequence length PE150 bp.

To evaluate the shortened adapters and redesigned barcodes, we constructed four *M*iddRAD sub-libraries according to protocol B for 40 offsprings of a *D. latiflorus* F1 population and sequenced the final library with a single illumina HiSeq X ten lane (PE150 bp). The coefficient of variation (CV = standard deviation/mean) of data generated by each barcode and each sub-library were analyzed to evaluate the newly designed barcodes, indexes and shortened P1 adapters.

### Sequence quality analysis, SNP calling and genotyping

Raw reads were demultiplexed by process_radtags program in Stacks software version 1.24 [30, 31]. Average sequence quality per read and GC–content were checked using FastQC version 0.11.3 [32]. Adapter reads were searched by Cutadapt 1.9.1 [33]. Reads containing correct restriction sites in read1 and read2 were obtained by searching restriction sites sequences in the raw reads respectively. Clean data were produced by removing the adapter reads and reads with ambiguous or low quality (below a Phred score of Q10) bases. To determine the mapping ratio of sampled reads to the genome, clean reads of *O. sativa*, *Z. mays* and *A. semialata* were mapped onto the rice, maize and sorghum genome scaffolds, CDS-DNA and repeats region respectively, while clean reads of temperate bamboo individuals onto the *P. edulis* reference genome, CDS-DNA region and repeats region and reads of tropical bamboo individuals onto the *D. latiflorus* survey genome (Zhenhua Guo et al. unpublished data) with bowtie [34]. Rice, maize and sorghum genome scaffolds, CDS-DNA and repeats region were downloaded from Plantgdb [35] while *P. edulis* reference genome, CDS region, and repeats region were downloaded from BambooGDB [36]. To obtain the number of tags, clean reads (we only used read1 for analysis) of all individuals were first trimmed 140 bp (when read length is PE150) and clustered with ustacks/pstacks program, then the reducing efficiency was determined by calculating the percentage of total tag length in total nuclear genome length.

To estimate the performance of *Mi*ddRAD protocol, tags of rice and maize produced by empirical sequencing results were compared with those predicted from in silico analysis to show how actual data meet the in silico expectations.

In order to identify SNP markers and genotypes for inferring phylogeny of three woody bamboo species, the Stacks software pipeline was implemented for the processing of Illumina sequence read data and screening SNPs that are fixed-within a species while vary among different species. Sequence trimming was first performed using process_radtags program to remove adapter reads and reads with bases below a Phred score of Q10 within a 15 bases sliding window. Clean sequences were truncated to a final length of 140 base pairs (excluding the barcode but containing enzyme recognition site) prior to clustering. For each sample, the ustacks program was used to merge short-read sequences into tags/loci using removal algorithm and deleveraging algorithm (−m10, −M3). Then a catalog was built from all samples by the cstacks program (−n5). Tags from each sample were matched against the catalog to determine alleles with sstacks program and the populations program was used to output SNPs in Phylip format. The minimum number of taxa required for an informative unrooted phylogenetic tree is three. The major parameters m (minimum number of identical reads required to form a stack), M (maximum number of nucleotides mismatches allowed between stacks before fusing stacks into a locus) and n (number of mismatches allowed between loci in the catalog) were tuned to get the matrix with a variable number of SNPs. Furthermore, to validate the genotyping accuracy, we presented linkage map results of one *D. latiflorus* F1 mapping population (Guoqian Yang et al., unpublished data) according to *Mi*ddRAD protocol and 55 genotypes (eight markers, seven individuals and one genotype was missing during the SNP calling pipeline) were randomly selected and verified by independent Sanger sequencing.

### Phylogenetic tree construction of three woody bamboo species

We inferred ML phylogenies for each data matrix using RAxML version 8.0.0 [37]. ML searches were conducted in RAxML with the GTRGAMMAI model for sequence data, and a rapid bootstrapping analysis with 100 bootstrap replicates was conducted. Phylogenetic accuracy was determined by comparing inferred trees with published reference phylogenies. The reference phylogeny for woody bamboos from [38, 39], was estimated using parsimony analyses (MP) and Bayesian inference (BI).

## Results

### Choosing universal restriction enzyme pairs across angiosperm plants

**Fig. 2:**
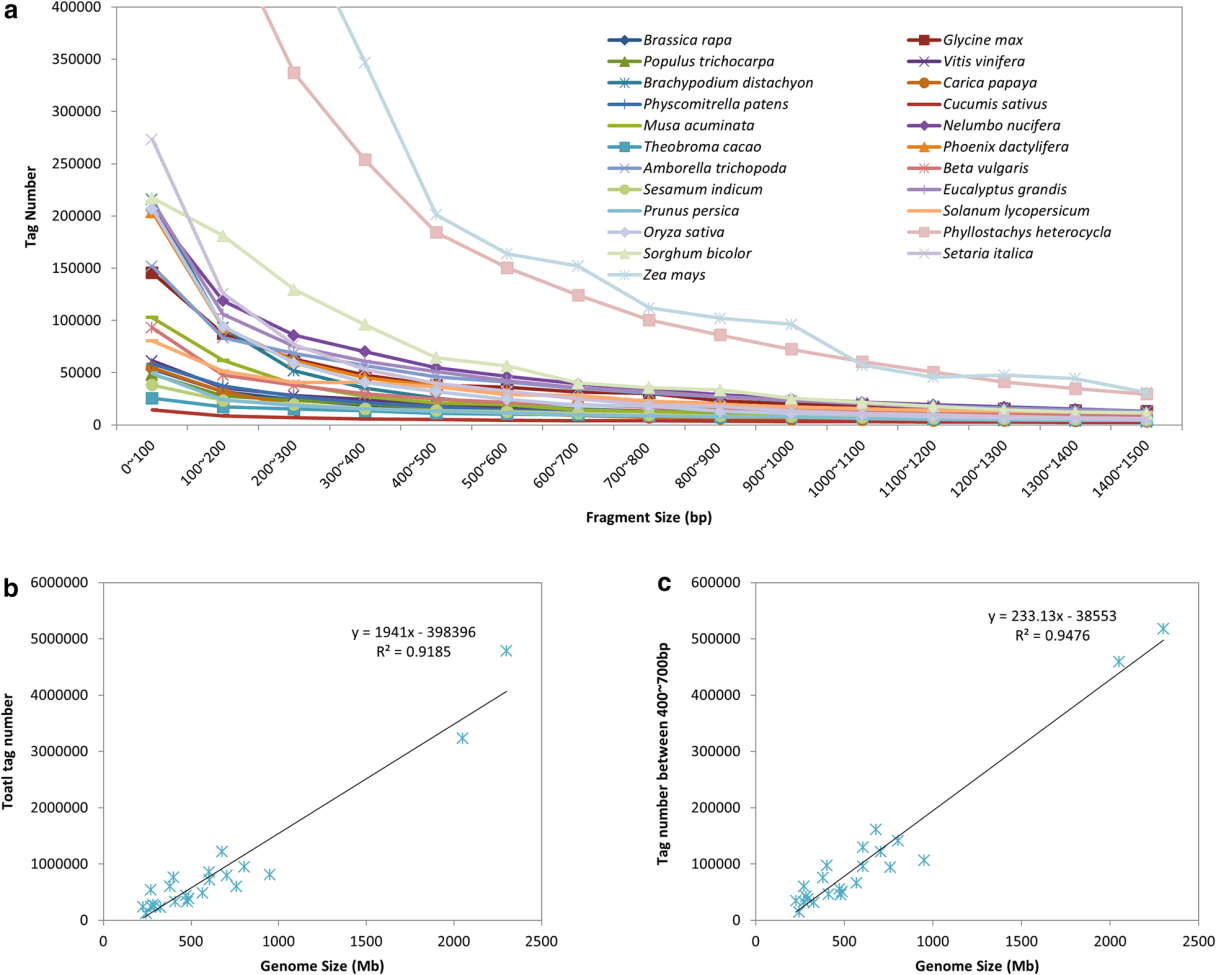
In silico digestion genome sequences of 23 plant species by *AvaII* + *MspI*. The *line graph* shows tag number at every 100 bp interval from 0–1500 bp (**a**). Both total tag number (**b**) and tag number within 400–700 bp (**c**) are correlated positively with genome size

Six combinations of endonucleases identifying 4–8 nucleotide bases were tested on genomes of 23 plant species (covering 17 families of angiosperm and one family of bryophyta). The ideal combination should be able to generate a consistently higher number of sequence-able fragments across species. The four bases + eight bases enzyme pair and six bases + eight bases enzyme pair usually produced a few thousand fragments, which were far from the requirement for large-scale genotyping (Table 1). However, the four bases + four bases enzyme pair produced up to ~23,112,695 fragments which made it difficult to control error in practice and required deep sequencing depth. Meanwhile, the four bases + six bases enzyme pair generally produced 32,319–886,527 fragments which made it easy for people to obtain a sufficient number of fragments without sequencing a large amount of data. *PstI* + *MspI* which was used in the original two-enzyme GBS protocol performed well in most plants, but only produced 3791 fragments between 400–700 bp for *Cucumis sativus* and 8258 fragments for *Carica papaya* (Table 2). *EcoRI* + *MspI* which was used in the original ddRAD protocol performed better than *PstI* + *MspI* in any simulated species by producing thousands or more tags, but only 8173 fragments fell into within 400–700 bp for *C. sativus*, which could not meet the demand for more tags in some studies. We found *AvaII* + *MspI* enzyme pair was superior to both of *EcoRI* + *MspI* and *PstI* + *MspI*. This enzyme pair could produce at least 13,958 segments between 400–700 bp in the 23 simulated species and was predicted to provide sufficient tags across diverse plant species. The largest genome (*Z. mays*, 2300 Mb) could produce 4,784,940 tags with 517,204 tags between 400–700 bp while even the smallest genome (*Prunus persica*, 226.6 Mb) could produce 237,185 tags with 34,514 tags between 400–700 bp (Fig. 2a; Table 2). Both of the enzymes are common enzymes with AvaII identifying 4.5 bases and MspI identifying four bases which are different from the combination with a common enzyme and a rare enzyme adopted in ddRAD or two-enzyme GBS. Correlation analysis showed that the total tag number is correlated positively with genome size with R^2^ = 0.9185 and tag number between 400–700 bp is correlated positively with genome size as well with R^2^ = 0.9476 (Fig. 2b, c). So once we get to know the genome size of one plant, the tag number produced could be estimated and the expected tag number could be tuned by selecting a proper size range. *EcoRI* + *MspI* and *PstI* + *MspI* could also be taken into consideration when designing a ddRAD project as they may produce hundreds to thousands of markers across a wide range of plant species and the total tag number or tag number between 400–700 bp is correlated positively with genome size as well (Additional file 2: Figure S2). After conducting the above simulations, we built ddRAD libraries with the *AvaII* + *MspI* enzyme pair. Fragments between 400–700 bp are highly recommended for their high sequencing efficiency on illumina system. Optimization of the ratio of sample DNA to the adapters is not required when the genome size is less than 20 Gb because we have added excess adapters in our protocol which could make each fragment be ligated with corresponding adapters (Adapter P1 contains about 3 × 10^12^ molecules while Adapter P2 contains about 6 × 10^12^ molecules). As average genome size of the angiosperm is 5.79 Gb while bryophyte is 0.66 Gb according to Plant DNA C-values Database at Kew [40], we believe that this combination of two common endonucleases may be applied to diverse plant species only by tuning the size selected.

**Table 1:** Total number of fragments produced by in silico digestion of 23 species

Species	*SbfI* + *EcoRI*	*SbfI* + *MlucI*	*NlaIII* + *MlucI*	*AvaII* + *MspI*	*EcoRI* + *MspI*	*PstI* + *MspI*
*Brassica rapa*	2352	2795	3,710,380	271,860	88,220	70,512
*Glycine max*	7498	9006	16,747,131	803,332	306,391	162,398
*Populus trichocarpa*	4876	5769	7,169,615	329,150	147,647	97,378
*Vitis vinifera*	4994	5903	8,028,433	380,967	162,506	91,994
*Brachypodium distachyon*	12,306	14,912	3,237,571	535,195	106,924	166,584
*Carica papaya*	4592	5255	4,221,765	245,762	99,163	59,272
*Physcomitrella patens*	4786	5413	7,434,539	329,065	144,069	102,802
*Cucumis sativus*	1450	1737	3,342,214	116,754	62,340	32,319
*Musa acuminata*	6599	7483	5,467,255	433,922	170,591	129,105
*Nelumbo nucifera*	9469	10,906	10,160,790	944,853	308,661	164,996
*Theobroma cacao*	3376	3979	6,027,724	226,779	114,082	80,088
*Phoenix dactylifera*	8978	10,334	7,341,396	716,454	218,488	177,248
*Amborella trichopoda*	11,702	13,558	10,008,594	787,796	234,856	120,412
*Beta vulgaris*	6458	7804	7,904,182	483,300	199,616	103,224
*Sesamum indicum*	3412	4135	4,915,092	247,403	113,511	78,634
*Eucalyptus grandis*	8364	9943	9,894,486	849,552	307,001	174,810
*Prunus persica*	3116	4351	3,505,137	237,185	90,631	67,280
*Solanum lycopersicum*	15,086	18,005	12,249,900	596,412	255,692	124,564
*Oryza sativa*	10,042	11,855	4,881,316	595,046	138,534	171,045
*Phyllostachys edulis*	51,097	58,916	23,112,695	3,233,281	734,959	707,912
*Sorghum bicolor*	21,528	23,978	8,200,062	1,217,504	301,472	329,922
*Setaria italica*	15,347	17,861	4,683,693	757,165	145,882	225,478
*Zea mays*	85,902	2797	21,096,385	4,784,940	807,008	886,527

**Table 2:** Number of fragments between 400–700 bp produced by in silico digestion of 23 species

Species	*SbfI* + *EcoRI*	*SbfI* + *MlucI*	*NlaIII* + *MlucI*	*AvaII* + *MspI*	*EcoRI* + *MspI*	*PstI* + *MspI*
*Brassica rapa*	132	287	42,400	42,261	14,803	11,512
*Glycine max*	521	524	51,073	105,780	42,596	18,519
*Populus trichocarpa*	441	226	20,508	45,611	23,272	13,128
*Vitis vinifera*	386	383	32,588	50,912	21,822	11,769
*Brachypodium distachyon*	796	1948	50,586	60,014	18,552	21,701
*Carica papaya*	276	235	19,405	32,969	14,169	8258
*Physcomitrella patens*	433	481	30,274	45,223	20,689	14,832
*Cucumis sativus*	103	83	10,113	13,958	8173	3791
*Musa acuminata*	389	913	51,225	55,068	26,123	17,209
*Nelumbo nucifera*	783	607	80,253	140,632	49,260	24,250
*Theobroma cacao*	242	209	16,355	31,259	17,838	12,145
*Phoenix dactylifera*	622	876	64,497	95,233	36,744	26,304
*Amborella trichopoda*	1285	972	61,985	121,796	35,348	16,701
*Beta vulgaris*	541	472	42,743	65,689	29,666	13,827
*Sesamum indicum*	277	152	15,658	37,530	17,857	11,229
*Eucalyptus grandis*	655	666	65,729	129,175	53,775	26,496
*Prunus persica*	274	123	14,466	34,514	14,194	9910
*Solanum lycopersicum*	2250	421	42,919	93,748	36,617	16,313
*Oryza sativa*	696	1451	61,098	75,621	23,537	24,080
*Phyllostachys edulis*	2309	7086	367,267	458,669	130,850	116,861
*Sorghum bicolor*	1923	2740	128,588	161,131	56,433	56,091
*Setaria italica*	896	2434	81,751	96,716	26,592	31,528
*Zea mays*	3344	289	521,797	517,204	122,210	131,141

### A comprehensive evaluation of the library quality and data quality

The performance of our protocols was evaluated from both the experimental results and data analysis results. From the experimental perspective, library concentration should meet the criteria for sequencing and fragments selected should be in the expected range. From data analysis perspective, the library should produce sufficient high-quality data for downstream analysis.

We first quantified concentration of the libraries and screened fragments distribution to evaluate the quality of protocol A and protocol B. Concentration of library A (constructed according to protocol A) was between 5–9 ng/ul, while concentration of library B (constructed according to protocol B) was between 20–30 ng/ul, both of which could meet the requirements for Illumina sequencing. Fragments distribution of library A and library B screened by the agarose gel electrophoresis is well within the expected range (Additional file 2: Figure S3a). Fragments distribution results for library B had been further confirmed by the Agilent 2100 Bioanalyzer (Additional file 2: Figure S3b) while library A got no peaks from Agilent 2100 because its concentration is lower than 10 ng/ul. Therefore, we believe that fragments distribution can be determined by using agarose gel electrophoresis instead of the highly sensitive but expensive Agilent 2100 Bioanalyzer. Both protocols could produce libraries that can be sequenced on the Illumina sequencing platform.

**Fig. 3:**
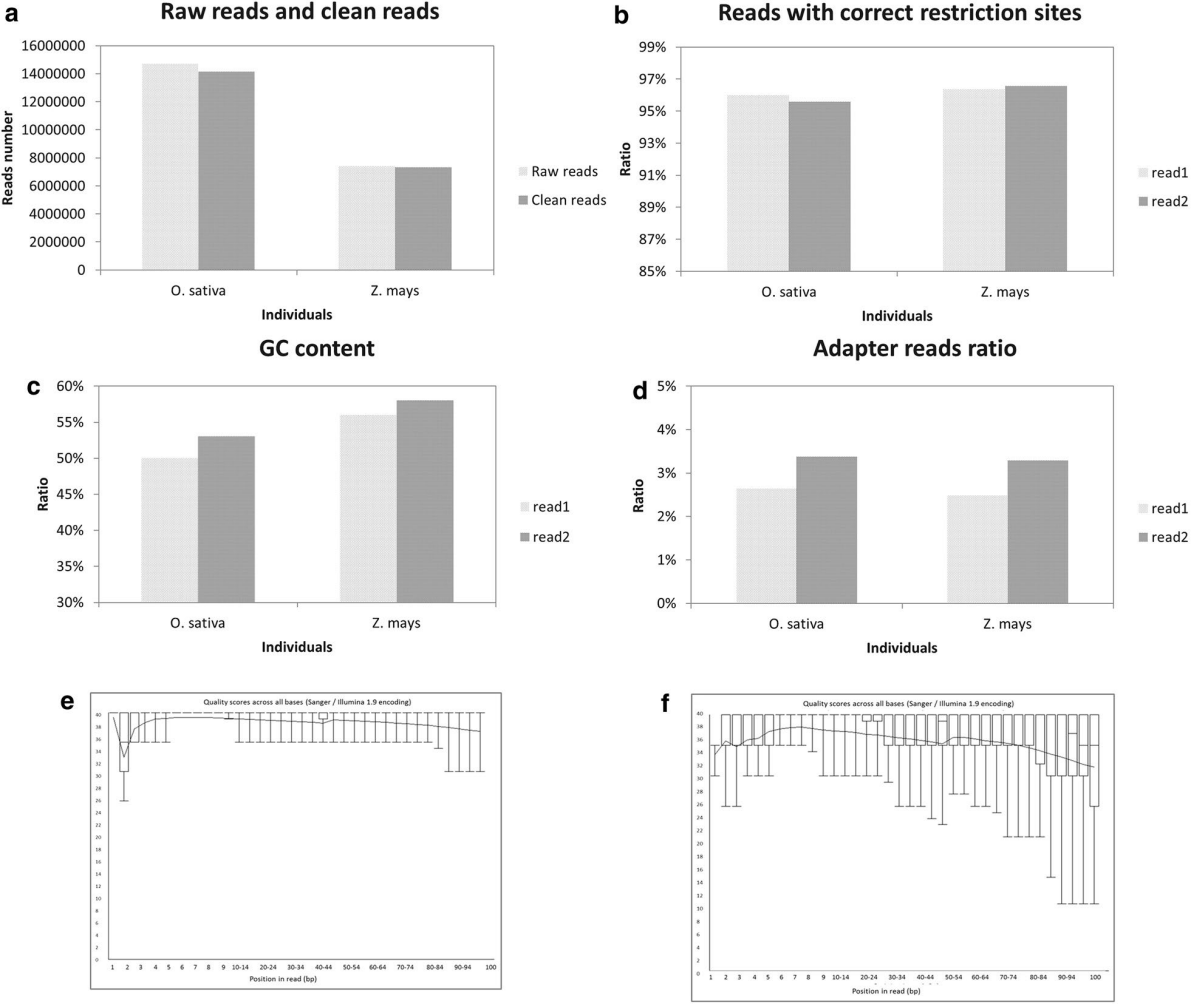
A comprehensive analysis of data produced by library B. This includes raw reads and clean reads number (**a**), correct restriction sites ratio (**b**), GC-content (**c**), adapter reads ratio (**d**), data quality of *O. sativa* read1 (**e**) and *O. sativa* read2 (**f**)

Then we conducted a comprehensive data analysis including data quality distribution, GC-content, adapter reads ratio and correct restriction sites ratio of both data produced by library A and library B. As for library A, *D. latiflorus* yielded a total of 2,890,217 raw reads (i.e. 578 Mb raw data) with 58 % GC-content; read1 containing correct restriction sites accounted 95.9 % of raw reads and read2 containing the correct restriction sites accounted for 94.8 %; read1 had a ratio of 49.3 % adapter reads while read2 had a ratio of 48.0 % adapter reads. *C. pallens* yielded a total of 3,146,515 raw reads (i.e. 629 Mb raw data) with 57 % GC-content; read1 containing correct restriction sites accounted 95.5 % of raw reads and read2 containing the correct restriction sites accounted for 94.5 %; read1 had a ratio of 40.3 % adapter reads while read2 had a ratio of 39.6 % adapter reads. Raw reads of *D. latiflorus* and *C. pallens* both had an average base Quality Score larger than 20. Furthermore, bases of restriction enzyme cutting site had an average base Quality Score larger than 30. As for library B, *O. sativa* yielded a total of 14,732,449 raw reads (i.e. 4.1 Gb raw data) with 51.5 % GC-content (Fig. 3a, c); read1 containing correct restriction sites accounted 95.80 % of raw reads and read2 containing the correct restriction sites accounted for 95.39 % (Fig. 3b); read1 had a ratio of 2.63 % adapter reads while read2 had a ratio of 3.37 % adapter reads (Fig. 3d). *Zea mays* yielded a total of 7,414,009 raw reads (i.e. 2.1 Gb raw data) with 57 % GC-content; read1 containing correct restriction sites accounted 96.18 % of raw reads and read2 containing correct restriction sites accounted for 96.37 %; read1 had 2.48 % adapter reads while read2 had 3.29 % adapter reads. Raw reads of *O. sativa* and *Z. mays* both had an average base Quality Score larger than 20 while bases of restriction enzyme cutting site had an average base Quality Score larger than 30 (Fig. 3e, f). To determine the mapping ratio of sampled reads to the reference genome, we mapped clean reads of rice and maize onto the rice and maize reference genome scaffolds, CDS-DNA and repeats region respectively. Overall scaffolds mapping rate was 82.5–90.66 %, reads mapping to the CDS-DNA accounts 2.38–2.83 % for maize and accounts ~19.50 % for rice. Yet reads mapping on the repeats region accounted for less than 11.00 % (Table 3).

**Table 3:** Summary of alignment statistics of sequencing data

Individual no.	Scaffolds (%)		CDS (%)		Repeats (%)	
	Read1	Read2	Read1	Read2	Read1	Read2
*Protocol A*						
*D. latiflorus*-*3*	63.21	61.35	–	–	–	–
*C. pallens*	15.83	13.95	3.31	3.10	0.49	0.39
*Protocol B*						
*Oryza sativa*	88.22	82.50	19.39	19.64	6.85	7.70
*Zea mays*	90.66	84.86	2.38	2.83	10.95	8.82
*P. edulis*	83.80	79.93	2.38	2.87	0.11	0.17
*A. semialata*	3.37	3.71	1.08	1.11	0.00	0.22
*D. latiflorus*-*1*	62.23	61.97	–	–	–	–
*D. latiflorus*-*2*	64.15	62.59	–	–	–	–
*P. rubicunda*	23.19	21.72	1.40	1.59	0.10	0.15
*P. vivax*	25.56	24.66	1.39	1.64	0.11	0.16

As CDS and repeats region were not available for *D. latiflorus* survey genome sequences, *Mi*ddRAD data of *D. latiflorus* individuals were only mapped to the assembled scaffolds

Per Bases Quality Score is a major index of the sequence quality, the higher the Quality Score, the lower probability of sequencing error occurs. Q20 and Q30 represent the sequencing error probability of 1 and 0.1 %. Illumina sequencing was found to favor the more GC-balanced regions, leading to few or no reads from the many GC-poor regions and GC bias can be introduced at several processes of Illumina sequencing, e.g. PCR amplification of the library, cluster amplification, and the sequencing step [41]. So if GC-content is around 50 %, we can conclude no bias exists in library preparation and sequencing process. Adapter reads ratio is the percentage of reads with adapters in raw reads and is an indicator of data quality. Adapter reads should be removed in the subsequent analysis. Through percentage of reads containing correct restriction sites, we can determine whether the enzyme digestion reaction works in the right way. Comprehensive analysis of data quality distribution, GC-content, adapter reads ratio and correct restriction sites ratio showed that both *Mi*ddRAD protocols could produce high-quality data (We did not compare our data with original ddRAD data as the original ddRAD protocol did not supply their raw data, but the quality of our data is self-explaining). However, protocol A produced too many (nearly half of raw reads) reads with adapters which indicated many short fragments may exist in the selected gel, so we did not continue testing protocol A in model plants and took protocol B as the final protocol of *Mi*ddRAD.

### Comparison of empirical and simulated data and inference tags origin from the genome

**Fig. 4:**
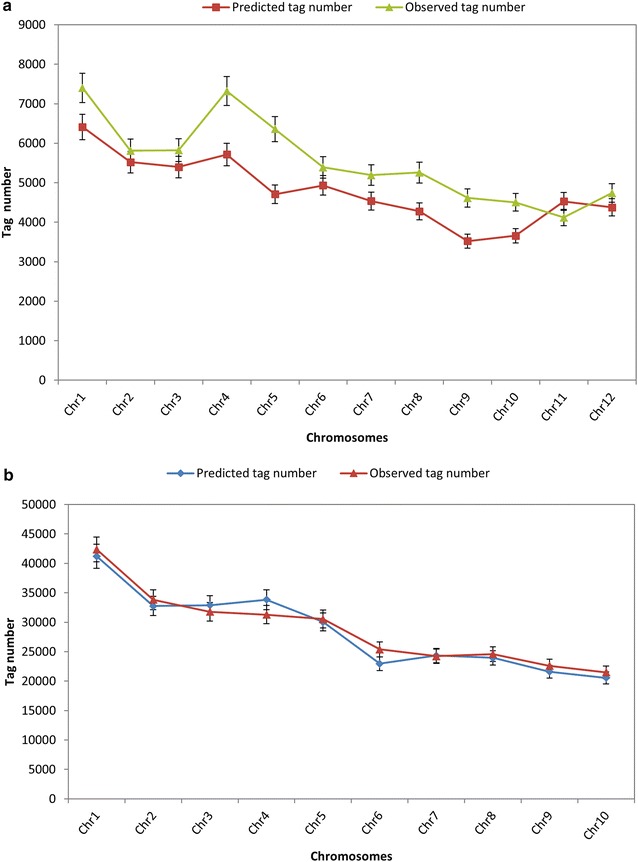
Comparison of the real sequencing data with in silico predicted results. **a** Each of the 12 rice chromosomes is expected to produce 3521–6414 fragments while each actually generates 4121–7404 tags with the Pearson correlation coefficient r = 0.8374. **b** The 10 maize chromosomes each is expected to produce 20,555–41,216 fragments while each is observed to generate 21,491–42,376 tags with the Pearson correlation coefficient r = 0.9792

A comprehensive evaluation of the protocol B was further conducted by comparison of the simulated data with the actual fragments we got. Clean data of rice and maize were clustered into tags using pstacks program. The number of tags obtained from rice was ~66,547 with an average depth of 212.58X while maize got 290,001 tags with an average depth of 25.30X (Table 4). The expected number of tags accounted for 86.54 and 97.99 % of the actual number respectively which is similar to the results done by Sun et al.(82.86 % for rice) [42]. Then we estimated the number of fragments distributed on 12 chromosomes of rice and 10 chromosomes of maize respectively. The actual number of tags obtained was compared to the expected data to test the degree of consistency (Fig. 4). We found each of the 12 rice chromosomes was expected to produce 3521–6414 fragments while each actually generated 4121–7404 tags with the Pearson correlation coefficient r = 0.8374. The 10 maize chromosomes each was expected to produce 20,555–41,216 fragments while each was observed to generate 21,491–42,376 tags with the Pearson correlation coefficient r = 0.9792. The actual and predicted data correlate well for maize while slightly worse for rice which is maybe due to the deviations introduced by cutting the gel. However, the observed tag number within the CDS region correlates better with expectation in rice than in maize (Table 4). It is noteworthy that while rice and maize own 39 and 85 % of repeat sequences respectively, only 15.83 and 31.44 % of tags fall into repeats region which indicates that the selected enzyme pair may be efficient in avoiding genome areas with highly repetitive DNA. It is supposed that this is because of a lack of restriction sites in some types of repetitive DNA as the two species contain more than 10 kinds of transposable elements respectively [43, 44]. As rice holds an average genome size of ~383 Mb and maize holds an average genome size of ~2300 Mb, the sampled tags only accounted for 1.77–2.43 % of the whole nuclear genome. In this sense, the efficiency of this reduced representation method on reducing genome complexity is reasonably high. From the overall mapping rate, we can infer that fragments should mainly fall into the intergenic region rather than CDS or repeats region. Our simplified approach effectively avoids repeats region in rice and maize which mainly includes transposons and retrotransposons that usually bring problems in determining orthologous fragments among different individuals.

**Table 4:** *Mi*ddRAD-seq data summary in rice and maize

Genome information	*Oryza sativa*	*Zea mays*
Genome size (Mb)	383	2300
% of repeats in genome	39.11	85.00
GC content (%)	43.56	46.83
Expected information		
Enzyme pairs	*AvaII* + *MspI*	*AvaII* + *MspI*
Expected RAD tag size range (bp)	460–680	500–680
Expected no. of RAD tags	60,925	284,179
Tags density per 100 kb	15.92	12.36
% of tags in CDS	25.10	3.04
Observed information		
Raw reads	14,732,449	7,414,009
Clean reads	14,146,516	7,337,556
Observed no. of tags	66,547	290,001
Tag average depth	212.58	25.30
Tags per 100 kb	17.38	12.61
Simplification ratio (%)	2.43	1.77
% of tags in CDS	31.49	1.15
% of tags in repeats	15.83	31.44

### Evaluation of protocol B on more plant species and genotypes validation

To test the universality of our protocol and the restriction enzymes on more plant species, we used Protocol B to construct libraries for *P. edulis* and *A. semialata* which represent two subfamilies of Gramineae (Bambusoideae and Panicoideae). We first inspected library quality. The ultimate library concentration was between 20–30 ng/ul, and fragments distribution was well within the expected range when screened by the agarose gel electrophoresis. Both libraries met the qualification for sequencing.

Then we conducted the data quality analysis of both *P. edulis* and *A. semialata* as shown in Table 5. Both *P. edulis* and *A. semialata* yielded more than 8 Mb raw reads (i.e. ~2 Gb raw data) with ~52 % GC-content; read1 containing correct restriction sites accounted 96.08–97.94 % of raw reads and read2 containing the correct restriction sites accounted for 94.85–96.39 %; read1 had 1.19–2.32 % adapter reads while read2 had 2.45–3.96 % adapter reads. Raw reads of *P. edulis* and *A. semialata* both had an average base Quality Score larger than 20, and Quality Score of bases of restriction enzyme cutting site was larger than 30.

**Table 5:** A comprehensive data analysis of *P. edulis* and *A. semialata*

Species	Raw reads no.	Percentage of adapter reads (%)		Percentage of reads with correct restriction sites (%)		GC content (%)	Clean reads no.	Tag no.	Average tag depth
		Read1	Read2	Read1	Read2				
*P. edulis*	8,142,517	1.19	2.45	97.94	96.39	52	8,045,315	128,803	30.18
*A. semialata*	14,651,272	2.32	3.96	96.08	94.85	53	14,299,253	98,869	96.18

Next, we mapped clean reads of *P. edulis* onto the *P. edulis* genome scaffolds, CDS-DNA, and repeats region and clean reads of *A. semialata* onto the sorghum genome scaffolds, CDS-DNA, and repeats region respectively. For *P. edulis*, overall scaffolds mapping rate was 79.93–83.80 %, reads hits to the CDS-DNA accounted 2.38–2.87 % (Table 3). Yet reads localization on the repeats region accounted for 0.11–0.17 %. For *A. semialata*, the overall scaffolds alignment rate was 3.37–3.71 %, reads hits to the CDS-DNA accounted 1.08–1.11 %. Yet reads localization on the repeats region accounted for 0.00–0.22 %. Overall scaffolds alignment rate is relatively low for *A. semialata* is because of the sequence differences between *A. semialata* and *Sorghum bicolor.*

At last, clean data of both individuals were clustered into tags. The number of tags obtained from *P. edulis* was 128,803 with an average depth 30.18X which correlated well with the expectation. While *A. semialata* got 98,869 tags, with an average depth of 96.18X which was not within expectation as the sorghum genome was used as the reference. Since *P. edulis* has an average genome size of ~2000 Mb and *A. semialata* has an average genome size of ~600 Mb (personal communications with Ms. Yang Yang), the tags we got accounted for 1.80 % of the *P. edulis* nuclear genome and 4.61 % of the *A. semialata* nuclear genome.

To verify how genotyping accuracy is maintained in the *Mi*ddRAD protocol, we have recently constructed a linkage map of *D. latiflorus* (Guoqian Yang et al., unpublished data) according to *Mi*ddRAD protocol and got a high-quality map of 2365 markers with an average map distance 1.56 cM. The 36 linkage groups generated were corresponding to the 36 haploid chromosomes of *D. latiflorus* and 52 of the 55 selected genotypes (94.55 %) were agreed with independent Sanger sequencing results (Additional file 2: Table S3). We believe that genotypes from *Mi*ddRAD-seq derived data should be of high genotyping accuracy as the fundamental of constructing a high-quality linkage map with tight map distances are the correct genotypes of most markers/loci [22, 45].

### Evaluation of shortened adapters and new barcodes

**Fig. 5:**
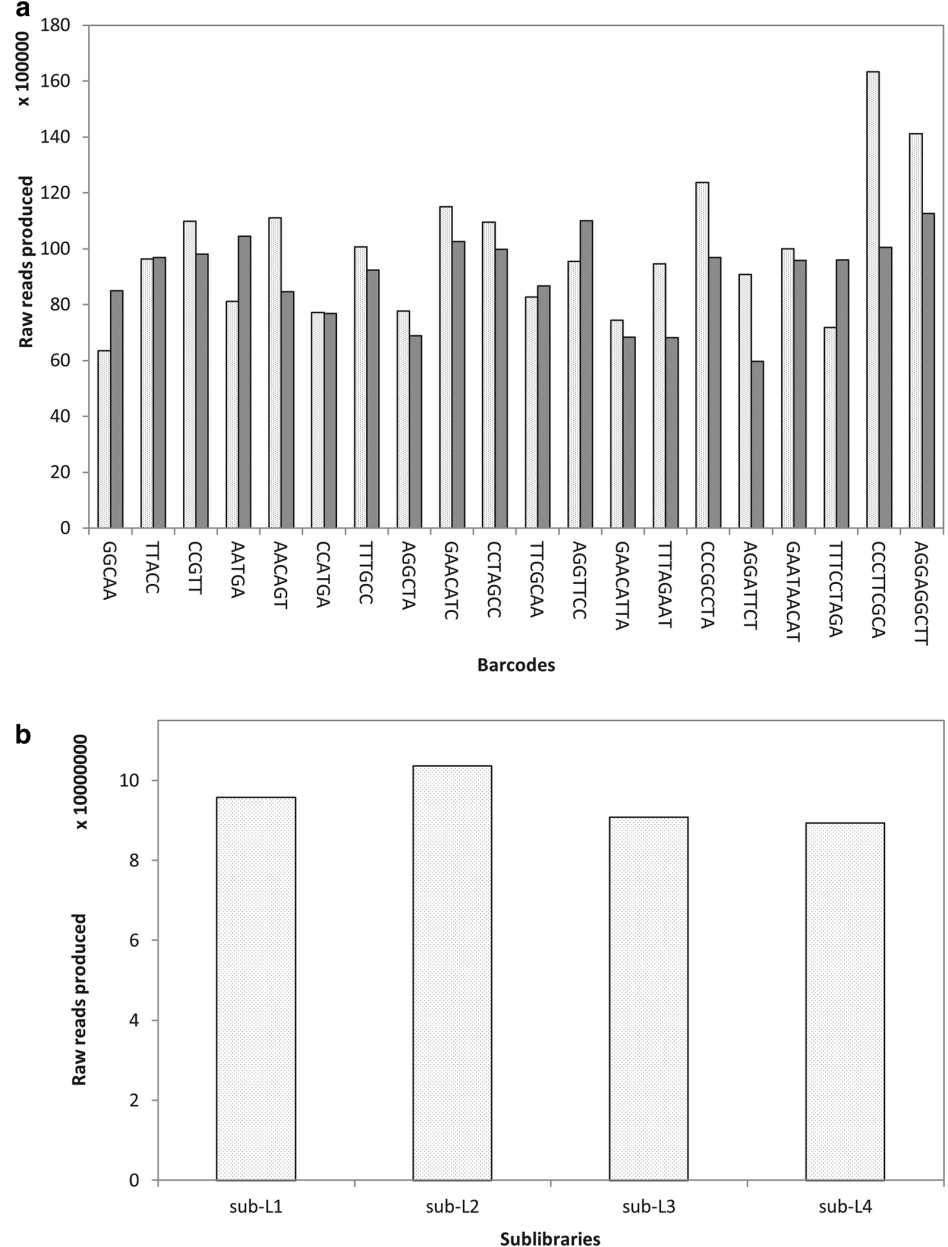
Histograms of data generated by each barcode and sub-library. **a** Distribution of reads across 40 barcoded samples in a single lane for the *D. latiflorus* F1 population. Each barcode was used twice. *White bars* represent data generated by each barcode the first time and *black bars* represent data generated by each barcode the second time. **b** Distribution of reads number across four indexed sub-libraries for the *D. latiflorus* F1 population

To evaluate the shortened adapters and redesigned barcodes, we constructed four *Mi*ddRAD sub-libraries containing 40 *D. latiflorus* individuals and sequenced the final library with a single Illumina lane. We used the double index strategy to distinguish each individual, which means each individual was identified by a unique barcode and index as the original ddRAD protocol implemented. We performed analysis of data generated by each barcode and found that each barcode and adapter could produce a relatively large amount of data with average 9,451,891 reads and CV value 0.0021–0.2381 (Fig. 5a). In addition, each sub-library could produce comparable amounts of data with a mean of 94,946,435 reads and CV value 0.0587 (Fig. 5b). This suggests that the newly designed barcodes and shortened P1 adapters are of high efficiency.

### Phylogenetic tree construction of three bamboo species

**Fig. 6:**
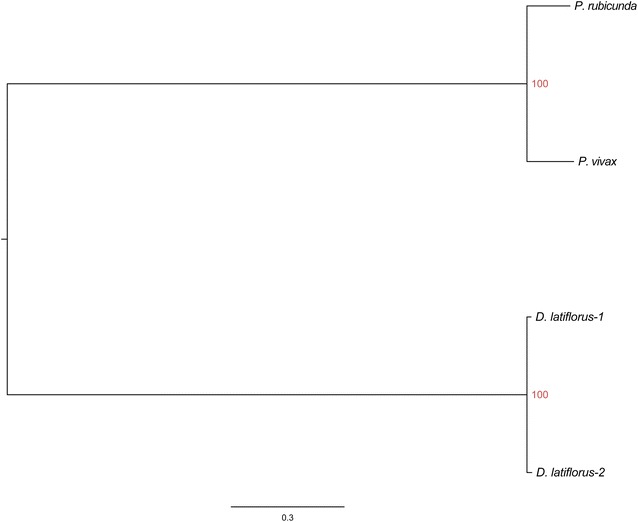
Maximum likelihood phylogenetic reconstruction of three bamboo species. Two clades are formed: the first contains the genus *Dendrocalamus*. *D. latiflorus* individual 1 is sister to *D. latiflorus* individual 2. In the second clade, *P. rubicunda* is sister to *P. vivax*, which themselves form a monophyletic clade

Maximum likelihood phylogenetic reconstruction is fully resolved with high support for all clades (2532 SNPs) (see Fig. 6). Two clades were found: the first contains the genus *Dendrocalamus* (100 % Bootstrap). *D. latiflorus* individual 1 is sister to *D. latiflorus* individual 2. In the second clade, *P. rubicunda* is sister to *P. vivax* (100 % Bootstrap), which themselves form a monophyletic clade (100 % Bootstrap). The relationships between two genera are well resolved and the topology of the two genera in our tree agrees well with current taxonomy [38, 39]. One additional RAxML analysis using alternatives data set (1005 SNPs) from Stacks analyses displayed identical topology and minor changes in branch lengths (Additional file 2: Figure S4).

## Discussion

In this study, we tested the universality of several commonly used enzyme pairs across the angiosperm plants, simplified the ddRAD protocol and reduced the overall costs. *Mi*ddRAD library construction protocol has optimized the following areas compared with original ddRAD protocol. (1) *Mi*ddRAD protocol tests the universality of several commonly used enzyme pairs and three pairs of restriction endonucleases are maybe universal in digesting plant genomic DNA. We recommend *AvaII* + *MspI* > *EcoRI* +*MspI* > *PstI* +*MspI* when designing plant ddRAD projects; (2) In *Mi*ddRAD protocol several expensive consumables and apparatuses are replaced by conventional experimental apparatuses, for example, the magnetic beads purification method is replaced by a simple column purification to get rid of the dependence on the magnet, DNA fragments are selected by cutting low melting point agarose gel rather than the automatically select device Pippin-Prep, and low melting point agarose gel electrophoresis is used to screen fragments distribution instead of an expensive Agilent 2100 Bioanalyzer; (3) *Mi*ddRAD removes 3 steps in ddRAD protocol, namely purifying the enzyme-digested products, quantifying the DNA concentration before ligation and purifying ligation products after pooling samples, all of which simplify the process of constructing a library; (4) In *Mi*ddRAD protocol original P1 adapters are shortened from 37 to 25 bp (barcode is assumed to be of 5 bp length), which will reduce the cost of the synthesizing adapter oligos partially; (5) In *Mi*ddRAD a new barcode-adapter system containing 20 pairs of barcodes varying in length were devised, which can be used with integer times (20 * n), rather than the original 48 kinds of barcode with equal length. This will not only reduce the cost of synthesizing DNA oligos but also increase the flexibility for projects with different samples and help improve the quality of bases near the restriction site. The comparison of *Mi*ddRAD with most commonly used RAD and GBS sequencing methodologies and associated costs are listed in Additional file 2: Table S4.

Since the thorough library construction process minimizes the purification times, random DNA loss is greatly reduced. The highly simplified process allows library preparation be accomplished with as low as 50 ng genomic DNA. Meanwhile, we found that reducing the two purification steps did not reduce the quality of the data by sequence quality analysis. Through data analysis of 40 individuals from a single lane, omitting the step quantifying DNA concentration of each individual before pooling samples does have influence on the amount of data among each individual (CV = 0.2146) but in our experience even if quantifying DNA concentration of each individual, pooling equal quantity DNA of each individual is still impossible as different volumes of liquid may adhere to the tips when using the pipette. As adequate data (>6 M reads) could be generated for each individual, we suggest deleting this quantifying step as the GBS protocol does (CV = 0.23) [16]. The redesigned adapters and variable length barcodes have high recognition efficiency on various individuals and could produce high-quality data which is similar to the results Burford et al. got [27]. Some people may worry that the combination of restriction endonucleases may easily cut the repeats region with high GC-content as is shown in maize about 10 % of reads fall into repeats region. Nevertheless, we still have enough reads left for analysis. Researchers may strictly follow the protocol without re-selecting novel combination of enzymes (but have to adjust the size-selection range) if they do not want to invest too much on pilot experiments. As synthetic adapters can be used in diverse plant species and transferred across labs, our protocol will greatly reduce the overall costs.

A possible drawback of our method is that degraded DNA will not produce adequate data because once one of the enzyme sites was impaired the whole tag will be lost. Nonetheless, as long as the DNA provided shows a clear major band when detected by the agarose gel electrophoresis, it could usually generate sufficient amount of data for analysis. In addition, the final library may be a pool of tens to a hundred of samples and we only designed 20 kinds of barcodes, so it is inevitable to cut gel several times when performing the procedure. In order to maintain the consistency of the selected fragments, electrophoresis conditions must be strictly controlled, and practice cutting the gel is needed before the formal experiment begins. Besides, electrophoresis time should be long enough (1–2 h) to prevent that size selection maybe ‘leaky’.

To demonstrate the applicability of *Mi*ddRADseq-derived markers in no-model species, we used *Mi*ddRAD data to resolve phylogenetic relationships of two woody bamboos genera, *Dendrocalamus* and *Phyllostachys*. *Dendrocalamus* is a tropical woody bamboo genus while *Phyllostachys* belongs to temperate woody bamboos. Our tree is congruent well with the current taxonomy. In comparison to previous studies in this clade, which used chloroplast regions [39] or nuclear DNA regions [38], the ddRAD data set is prominent for its simpleness in getting an amount of data (over 200 loci in the smallest data set). Though RAD-seq has been demonstrated to be feasible in clades as old as 40–60 million years with simulated RAD tags of Drosophila [46] and bona fide sequence of American oak [47], RAD sequences are usually considered useful for phylogenetic reconstruction in younger clades in which sufficient numbers of orthologous restriction sites are retained across species [46]. However, RAD-seq is now receiving increased attention at deeper evolutionary time scales, such as genus- or family-level phylogenetics even the problem of efficiently obtaining sequence data across many individuals exists [48]. Our study demonstrates the utility of *Mi*ddRAD data for reconstructing phylogenetic relationships in a group that spans 43–47 million-year-old divergences [49]. What we should bear in mind is that the performance of RAD or *Mi*ddRAD depends in part on the level of divergence between species. Determining orthologous RAD tags between samples should also be taken carefully in the future phylogenetic analysis with RAD sequencing [50]. The data set and analyses we provide here are a novel step forward in the use of ddRAD data to address questions in woody bamboo phylogenetic reconstruction. We show that it is possible to assemble genome-wide RAD-tags into phylogenetic matrices without the use of a reference genome.

## Conclusions

In this study, we first tested the universality of several commonly used enzyme pairs across 23 plant species and found *AvaII* + *MspI* enzyme pair produced a consistently higher number of fragments in a broad range of angiosperm plant species. Then we simplified the ddRAD protocol and designed a new barcode-adapter system that could reduce the overall costs. At last, we demonstrated the use of *Mi*ddRAD-seq data in resolving phylogenetic relationships of two woody bamboos genera. This protocol could help botanist quickly get ideal experimental data at a relatively low cost and without being specially trained. We expect that the protocol could be implemented efficiently nearly in any ordinary molecular laboratory without relying on large sequencing centers or next-generation sequencing companies.

## Additional files

10.1186/s13007-016-0139-1 Details of *Mi*ddRAD library constructing protocol. *Mi*ddRAD contains protocol A and protocol B and we take protocol B as the final protocol. A step-by-step tutorial was presented in this file.
10.1186/s13007-016-0139-1 A list of Additional Figures and Tables. **Figure S1.** Library preparation flowchart of *Mi*ddRAD protocol A. **Figure S2.** In silico digestion genome sequences of 23 plant species by *EcoRI* + *MspI* and *PstI* + *MspI*. **Figure S3.** Fragments distribution of library A and library B. **Figure S4.** Maximum likelihood phylogenetic reconstruction of three bamboo species. **Table S1.** Species adopted for in silico digestion and the corresponding genome size. **Table S2.** Restriction enzymes included in this study. **Table S3.** Independent Sanger sequencing for genotype validation of *Mi*ddRAD-seq genotyping. **Table S4.** Comparison of most commonly used RAD and GBS sequencing methodologies and associated costs.

## Abbreviations

RAD-seq: restriction-site associated DNA sequencing
ddRAD: double digest restriction associated DNA
*Mi*ddRAD: modified double digest restriction associated DNA
2b-RAD: IIB restriction endonucleases restriction-site associated DNA
GBS: genotyping by sequencing
Read1: forward read of pair-end fastq data
Read2: reverse read of pair-end fastq data
CDS: coding sequences
Repeats region: repetitive sequences in a genome
SNPs: single nucleotide polymorphism
Rare-cutter enzyme: a restriction enzyme with a recognition sequence which occurs only rarely in a genome
Common-cutter enzyme: a restriction enzyme with a recognition sequence which occurs frequently in a genome
Adapter: double-stranded product by annealing two oligos
Barcodes: short DNA sequence for identifying different individuals
