# Supplementary material for: Development of a universal and simplified ddRAD library preparation approach for SNP discovery and genotyping in angiosperm plants
Source: Plant Methods. 2016 Aug 4;12:39. doi: 10.1186/s13007-016-0139-1 (PMC4973087; doi:10.1186/s13007-016-0139-1)
Supplement: Supplementary file 1 — 10.1186/s13007-016-0139-1 Details of MiddRAD library constructing protocol. MiddRAD contains protocol A and protocol B and we take protocol B as the final protocol. A step-by-step tutorial was presented in this file. [file 13007_2016_139_MOESM1_ESM.doc]

***Mi*ddRAD protocol**

Guo Lab, Kunming Institute of Botany, CAS

12 Dec 2015

**Adapters, Barcodes and Primers**

***P1 Adapter*** is composed of the positive and negative strands,

Positive strand: 5’-TACACGACGCTCTTCCGATCTXXXXX-3’，

Negative strand: 5’-GWCYYYYYAGATCGGAAGAGCGTCGTGTA-3’.

XXXXX in the positive strand represent DNA barcode sequences. YYYYY in the negative-strand represent complementary sequences to DNA barcodes. W represents A or T.

There are a total of 20 DNA ***barcodes*** in Adapter P1 which are as follows,

| NO. | DNA barcode | NO. | DNA barcode |
| --- | --- | --- | --- |
| 1 | GGCAA | 11 | TTCGCAA |
| 2 | TTACC | 12 | AGGTTCC |
| 3 | CCGTT | 13 | GAACATTA |
| 4 | AATGA | 14 | TTTAGAAT |
| 5 | AACAGT | 15 | CCCGCCTA |
| 6 | CCATGA | 16 | AGGATTCT |
| 7 | TTTGCC | 17 | GAATAACAT |
| 8 | AGGCTA | 18 | TTTCCTAGA |
| 9 | GAACATC | 19 | CCCTTCGCA |
| 10 | CCTAGCC | 20 | AGGAGGCTT |

***P2 Adapter*** is composed of the positive and negative strand as well,

Positive strand: 5’-CGATAGATCGGAAGAGCCTCTTAGC-3’，

Negative strand: 5’-GTGACTGGAGTTCAGACGTGTGCTCTTCCGATCTAT-3’.

***Primers*** used in PCR amplification included primer sequences 1.1 and primer sequence 2.1, the nucleotide sequence of primers were as follows,

Primer sequences 1.1:

5’-AATGATACGGCGACCACCGAGATCTACACTCTTTCCCTACACGACGCTCTTCC-3’,

Primer sequences 2.1:

5’-CAAGCAGAAGACGGCATACGAGATNNNNNGTGACTGGAGTTCAGACGTGTGC-3’,

NNNNN in Primer sequences 2.1 represents index sequences that are used to distinguish different individuals which can adopt those Illumina recommends.

**Reagents and apparatuses required:**

***Reagents:***

AvaII (NEB or Promega), MspI (NEB or Promega), T4 DNA ligase (NEB or Vazyme), PCR phusion mix (NEB or Vazyme), Agarose (Biowest or BBI), DNA Ladder (SunShineBio), E.Z.N.A.® Gel Extraction Kit, E.Z.N.A.® Cycle Pure Kit.

***Apparatuses:***

Pipettes, Thermal Cycler, Thermostatic Water Bath, Centrifuge, Gel Electrophoresis Apparatus, Qubit 2.0 Fluorometer, NanoDrop ND1000 spectrophotometry (optional).

**Protocol A (Not recommended)**,

***Step1, genomic DNA extraction***

Young leaves of *D. latiflorus* and *C. pallens* were extracted using a modified CTAB method. After completion of DNA extraction 0.8% agarose gel electrophoresis was used to ensure the integrity of DNA and NanoDrop ND1000 spectrophotometry to give an indication of the concentration and cleanliness. Later, DNA was diluted to 40ng/μl.

***Step2, double enzyme digestion***

Two restriction enzymes AvaII (NEB) and MspI (NEB) were used to digest genomic DNA.

Digestion reaction system is as follows: 5.0μl genomic DNA (40ng/μl), 0.8μl AvaII (10，000U/ml), 0.4μl MspI (20,000U/ml) , 4.0μl 10 X CutSmart buffer, 29.8μl ddH_2_O, with a total volume 40.0μl. Digestion reaction conditions: digestion at 37°C for 4h; enzymes inactivation at 65°C for 20min; digestion products were preserved at 4°C.

***Step3, P1 and P2 adapters ligation***

P1 adapter (DNA barcodes adapter) and P2 adapter were ligated to the end of DNA fragments produced at the second step with T4 DNA ligase producing sequences in the form "P1 adapter -DNA inserts-P2 adapter". The ligation system is: 40.0μl digested products, 1.0μl Adapter P1 (5μM), 4.0μl Adapter P2 (10μM), 4.0μl 10XT4 ligation buffer, 0.5μl T4 DNA ligase, and 0.5μl ddH_2_O, with a total volume 50.0μl. Ligation reaction conditions: ligation at 23°C for 2h; enzymes inactivation at 65°C for 20min; ligation products were preserved at 4°C. Annealing adapters could follow the steps original ddRAD protocol adopted.

***Step4, Sample pooling***

Adapter-ligated fragments from the third step were pooled with a volume ratio 1: 1.

***Step5, purification of the pooled products with a spin column***

Column used for purification is the spin column E.Z.N.A.® Cycle Pure Kit provides.

[E.Z.N.A.® Cycle Pure Kit Protocol is as follows,

1. Transfer the samples into a clean 1.5 mL microcentrifuge tube. Add 5 volumes CP Buffer.

Note: Volume refers to the size of your PCR reaction.

2. Vortex to mix thoroughly. Briefly centrifuge to collect any drops from the inside of the lid. Insert a HiBind® DNA Mini Column into a 2 mL provided Collection Tube. Add the sample from Step 5 to the HiBind® DNA Mini Column. Centrifuge at maximum speed (≥13,000 g) for 1 minute at room temperature.

3. Discard the filtrate and reuse collection tube. Add 700μL DNA Wash Buffer. Centrifuge at maximum speed for 1 minute. Discard the filtrate and reuse collection tube. Repeat this step.

Note: DNA Wash Buffer must be diluted with ethanol before use.

4. Centrifuge the empty HiBind® DNA Mini Column at maximum speed for 2 minutes to dry the column.

Note: This step is critical for removal of trace ethanol that may interfere with downstream applications.

5. Transfer the HiBind® DNA Mini Column into a clean 1.5 mL microcentrifuge tube. Add 30μL Elution Buffer directly to the center of the column matrix. Let sit at room temperature for 2 minutes. Centrifuge at maximum speed for 1 minute. The product obtained is the DNA library to be sequenced. Store the library at -20°C.

Note: This represents approximately 80-90% of bound DNA. An optional second elution will yield any residual DNA, though at a lower concentration. ]

***Step6, enrichment of target DNA fragment by PCR amplification***

The pooled DNA fragments were as templates for PCR amplification to enrich DNA fragments. Primers were synthesized by BGI (Shenzhen) biotechnology Co., Ltd.

PCR amplification system is as follows：20.0μl mixed genomic DNA, 25.0μl PCR phusion mix (NEB or Vazyme), 1.0μl Primer 1.1 (10μM), 1.0μl Primer 2.1 (10μM), 3.0μl ddH_2_0, with a total volume 50.0μl. PCR reaction conditions: denaturing at 98°C for 3min; after which is the cycling procedure: denaturing at 98°C for 20s, annealing at 62°C for 30s, extending at 72°C for 30s, a total of 16 cycles; then extending at 72°C for 15min; hold at 4°C for preservation.

***Step7, Size selection of DNA fragments by agarose gel electrophoresis***

PCR products from the sixth step were loaded on a 2.0% agarose gel for electrophoresing. Electrophoresis buffer was fresh 1XTAE. The voltage was 4v/cm and electrophoresis time was 90min. We selected 600-700bp gel as the target fragments for sequencing.

***Step8, screen of fragments distribution and DNA library evaluation***

The library quality was detected by 2% low melting point agarose gel electrophoresis. The gel was run 90mins at a voltage of 120v.

***Step9, library quantification and sequencing***

Concentration of the library was estimated by Qubit 2.0 and the library was diluted to 10nM for sequencing. Sequencing platform was Illumina HiSeq 2000 with sequence length be PE101bp.

**Protocol B (Recommended),**

***Step1, genomic DNA extraction***

Young leaves of *P. viridi* and *P. japonica* were extracted using a modified CTAB method. After completion of DNA extraction 0.8% agarose gel electrophoresis was used to detect the integrity of DNA and NanoDrop ND1000 spectrophotometry was used to give an indication of the concentration and cleanliness. Later, DNA was diluted to 40ng/μl.

***Step2, double enzyme digestion***

Two restriction enzymes AvaII (NEB) and MspI (NEB) were used to digest genomic DNA.

Digestion reaction system is as follows: 5.0μl genomic DNA (40ng/μl), 0.8μl AvaII (10,000U/ml), 0.4μl MspI (20,000U/ml) , 4.0μl 10 X CutSmart buffer, 29.8μl ddH_2_O, with a total volume 40.0μl. Digestion reaction conditions: digestion at 37°C for 4h; enzymes inactivation at 65°C for 20min; digestion products were preserved at 4°C.

***Step3, P1 and P2 adapter s ligation***

P1 adapter (DNA barcodes adapter) and P2 adapter were ligated to the end of DNA fragments produced at the second step with T4 DNA ligase. The ligation system is: 40.0μl digested products, 1.0μl Adapter P1 (5μM), 4.0μl Adapter P2 (10μM), 4.0μl 10XT4 ligation buffer, 0.5μl T4 DNA ligase, and 0.5μl ddH_2_O, with a total volume 50.0μl. Ligation reaction conditions: ligation at 23°C for 2h; enzymes inactivation at 65°C for 20min; ligation products were preserved at 4°C. Annealing adapters could follow the steps original ddRAD protocol adopted. Optimization of the ratio of sample DNA to the adapters is not required because we have added excess adapters in our protocol which could make each fragment be ligated with corresponding adapters. Adapter P1 has about 3X10^12^ molecules while Adapter P2 has about 6X10^12^ molecules which are more than the DNA fragments produced (about 2.5X10^12^ if the average fragment size is 256bp). This protocol should also be suitable for gymnosperms with large genomes.

***Step4, Sample pooling***

Adaper-ligated fragments from the third step were pooled with a volume ratio 1: 1.

***Step5, Size selection of DNA fragments by agarose gel electrophoresis***

Products from the fourth step were loaded on a 2.0% agarose gel for electrophoresing. Electrophoresis buffer was fresh 1XTAE. The voltage was 4v/cm and electrophoresis time was 90min. We cut the size of 600-700bp gel as the target fragments for enrichment and PCR amplification.

***Step6, enrichment of target DNA fragment by PCR amplification***

DNA fragments recovered were as templates for PCR amplification to enrich target DNA fragments. Primers were synthesized by BGI (Shenzhen) biotechnology Co., Ltd.

PCR amplification system is as follows: 20.0μl mixed genomic DNA, 25.0μl PCR phusion mix (NEB or Vazyme), 1.0μl Primer 1.1 (10μM), 1.0μl Primer 2.1 (10μM), 3.0μl ddH_2_0, with a total volume 50.0μl. PCR reaction conditions: denaturing at 98°C for 3min; after which is the cycling procedure: denaturing at 98°C for 20s, annealing at 62°C for 30s, extending at 72°C for 30s, a total of 16 cycles; then extending at 72°C for 15min; hold at 4°C for preservation (The less PCR cycles the better when the final concentration is large than 10ng/μl) .

***Step7, purification of the PCR products with a spin column***

Column used for purification is the spin column E.Z.N.A.® Cycle Pure Kit provides.

***Step8, screen of fragments distribution and DNA library evaluation***

The library quality was detected by 2% low melting point agarose gel electrophoresis. The gel was run 90mins at a voltage of 120v.

***Step9, library quantification and sequencing***

Concentration of the library was estimated by Qubit 2.0 and the library was diluted to 10nM for sequencing. Sequencing was performed on the Illumina HiSeq 2000 instrument (Illumina Inc., San Diego, CA, USA) using the pair read, 101 nucleotide configuration.
