# Supplementary material for: Development of a universal and simplified ddRAD library preparation approach for SNP discovery and genotyping in angiosperm plants
Source: Plant Methods. 2016 Aug 4;12:39. doi: 10.1186/s13007-016-0139-1 (PMC4973087; doi:10.1186/s13007-016-0139-1)
Supplement: Supplementary file 2 — 10.1186/s13007-016-0139-1 A list of Additional Figures and Tables. Figure S1. Library preparation flowchart of MiddRAD protocol A. Figure S2. In silico digestion genome sequences of 23 plant species by EcoRI + MspI and PstI + MspI. Figure S3. Fragments distribution of library A and library B. Figure S4. Maximum likelihood phylogenetic reconstruction of three bamboo species. Table S1. Species adopted for in silico digestion and the corresponding genome size. Table S2. Restriction enzymes included in this study. Table S3. Independent Sanger sequencing for genotype validation of MiddRAD-seq genotyping. Table S4. Comparison of most commonly used RAD and GBS sequencing methodologies and associated costs. [file 13007_2016_139_MOESM2_ESM.ppt]

## Slide 1
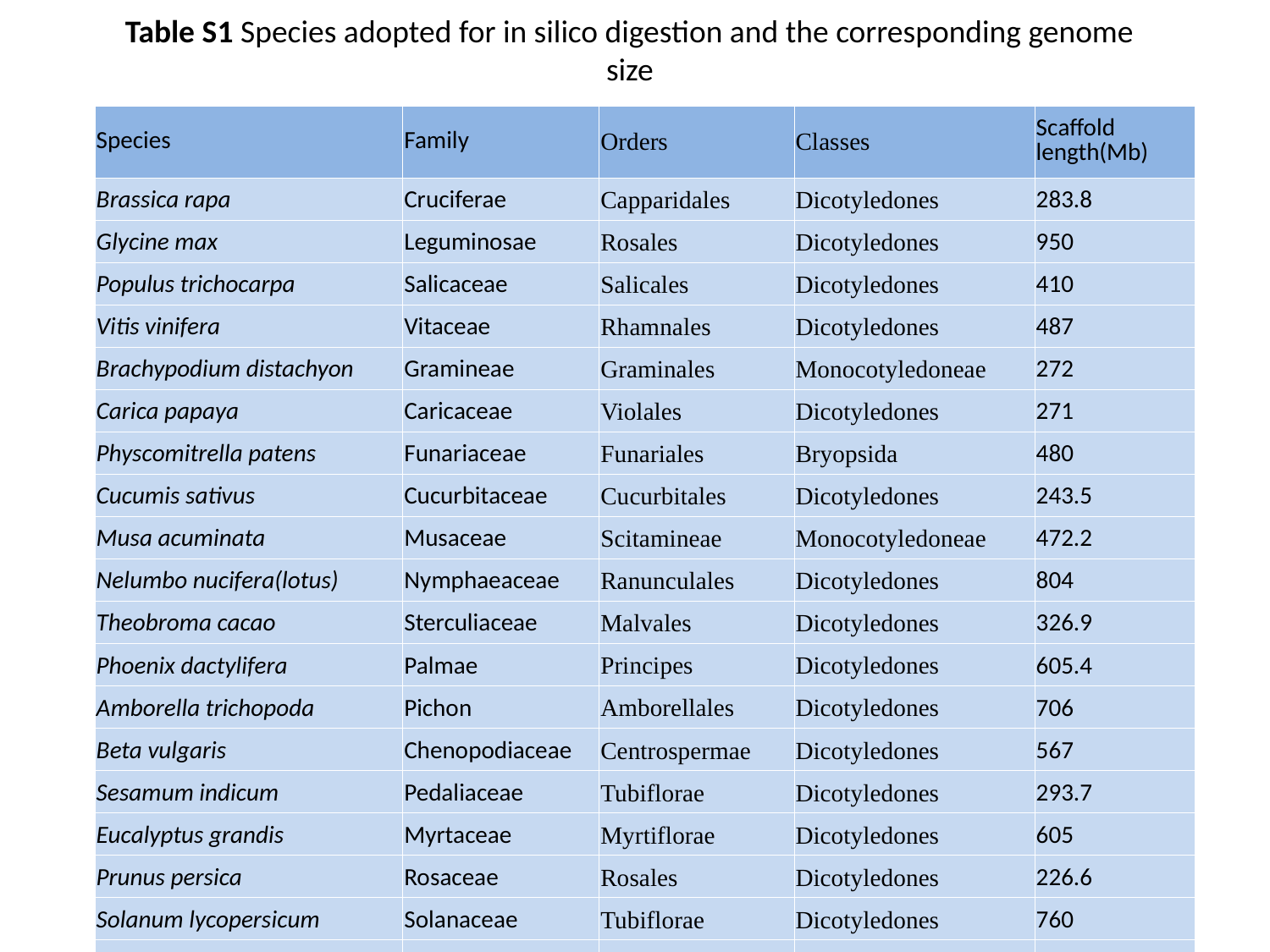

Table S1 Species adopted for in silico digestion and the corresponding genome size
| Species | Family | Orders | Classes | Scaffold length(Mb) |
| --- | --- | --- | --- | --- |
| Brassica rapa | Cruciferae | Capparidales | Dicotyledones | 283.8 |
| Glycine max | Leguminosae | Rosales | Dicotyledones | 950 |
| Populus trichocarpa | Salicaceae | Salicales | Dicotyledones | 410 |
| Vitis vinifera | Vitaceae | Rhamnales | Dicotyledones | 487 |
| Brachypodium distachyon | Gramineae | Graminales | Monocotyledoneae | 272 |
| Carica papaya | Caricaceae | Violales | Dicotyledones | 271 |
| Physcomitrella patens | Funariaceae | Funariales | Bryopsida | 480 |
| Cucumis sativus | Cucurbitaceae | Cucurbitales | Dicotyledones | 243.5 |
| Musa acuminata | Musaceae | Scitamineae | Monocotyledoneae | 472.2 |
| Nelumbo nucifera(lotus) | Nymphaeaceae | Ranunculales | Dicotyledones | 804 |
| Theobroma cacao | Sterculiaceae | Malvales | Dicotyledones | 326.9 |
| Phoenix dactylifera | Palmae | Principes | Dicotyledones | 605.4 |
| Amborella trichopoda | Pichon | Amborellales | Dicotyledones | 706 |
| Beta vulgaris | Chenopodiaceae | Centrospermae | Dicotyledones | 567 |
| Sesamum indicum | Pedaliaceae | Tubiflorae | Dicotyledones | 293.7 |
| Eucalyptus grandis | Myrtaceae | Myrtiflorae | Dicotyledones | 605 |
| Prunus persica | Rosaceae | Rosales | Dicotyledones | 226.6 |
| Solanum lycopersicum | Solanaceae | Tubiflorae | Dicotyledones | 760 |
| Oryza sativa | Gramineae | Graminales | Monocotyledoneae | 380 |
| Phyllostachys heterocycla | Gramineae | Graminales | Monocotyledoneae | 2050 |
| Sorghum bicolor | Gramineae | Graminales | Monocotyledoneae | 678.9 |
| Setaria | Gramineae | Graminales | Monocotyledoneae | 400 |
| Zea mays | Gramineae | Graminales | Monocotyledoneae | 2300 |

## Slide 2
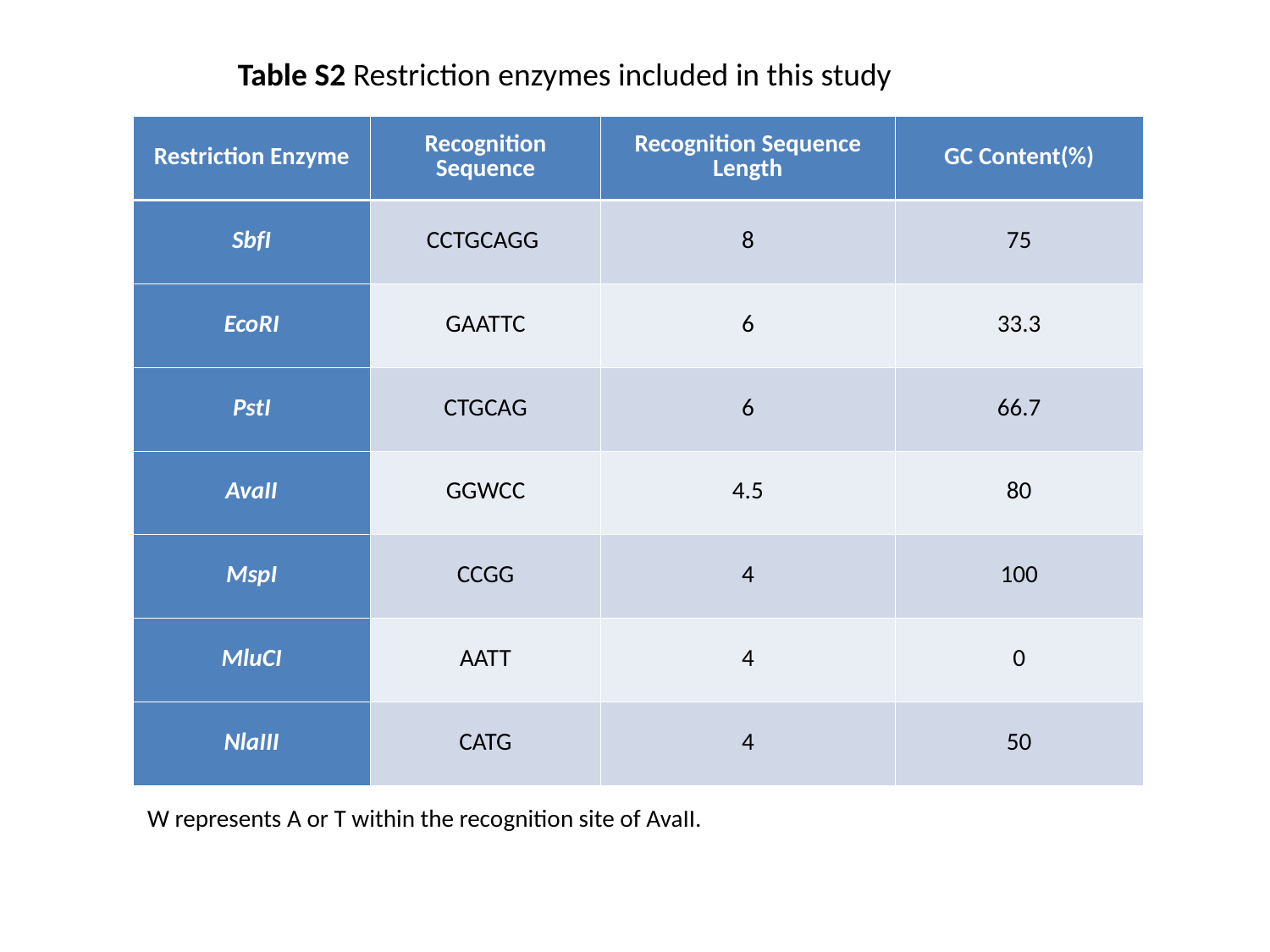

Table S2 Restriction enzymes included in this study
| Restriction Enzyme | Recognition Sequence | Recognition Sequence Length | GC Content(%) |
| --- | --- | --- | --- |
| SbfI | CCTGCAGG | 8 | 75 |
| EcoRI | GAATTC | 6 | 33.3 |
| PstI | CTGCAG | 6 | 66.7 |
| AvaII | GGWCC | 4.5 | 80 |
| MspI | CCGG | 4 | 100 |
| MluCI | AATT | 4 | 0 |
| NlaIII | CATG | 4 | 50 |
W represents A or T within the recognition site of AvaII.

## Slide 3
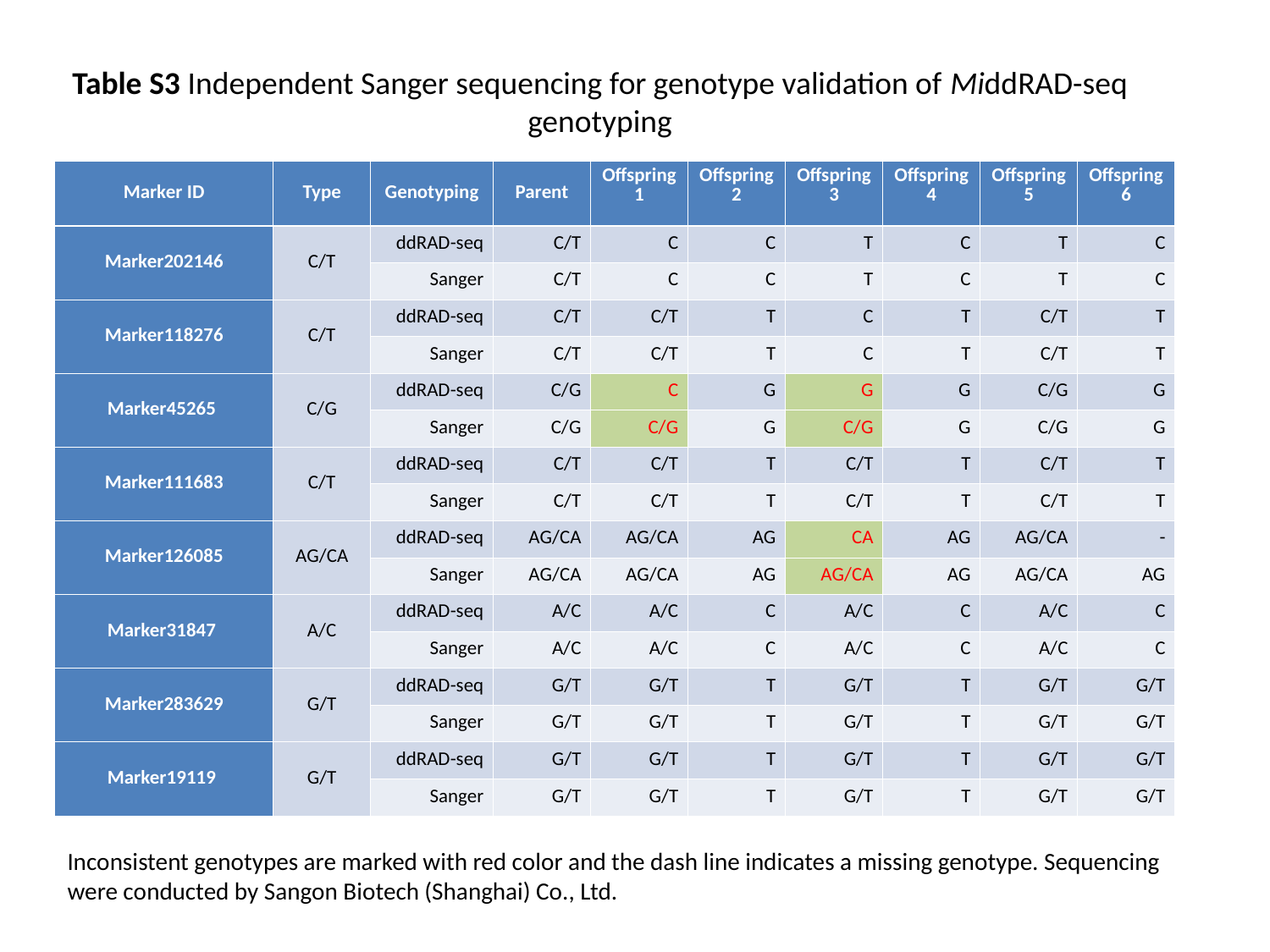

Table S3 Independent Sanger sequencing for genotype validation of MiddRAD-seq genotyping
| Marker ID | Type | Genotyping | Parent | Offspring1 | Offspring2 | Offspring3 | Offspring4 | Offspring5 | Offspring6 |
| --- | --- | --- | --- | --- | --- | --- | --- | --- | --- |
| Marker202146 | C/T | ddRAD-seq | C/T | C | C | T | C | T | C |
| | | Sanger | C/T | C | C | T | C | T | C |
| Marker118276 | C/T | ddRAD-seq | C/T | C/T | T | C | T | C/T | T |
| | | Sanger | C/T | C/T | T | C | T | C/T | T |
| Marker45265 | C/G | ddRAD-seq | C/G | C | G | G | G | C/G | G |
| | | Sanger | C/G | C/G | G | C/G | G | C/G | G |
| Marker111683 | C/T | ddRAD-seq | C/T | C/T | T | C/T | T | C/T | T |
| | | Sanger | C/T | C/T | T | C/T | T | C/T | T |
| Marker126085 | AG/CA | ddRAD-seq | AG/CA | AG/CA | AG | CA | AG | AG/CA | - |
| | | Sanger | AG/CA | AG/CA | AG | AG/CA | AG | AG/CA | AG |
| Marker31847 | A/C | ddRAD-seq | A/C | A/C | C | A/C | C | A/C | C |
| | | Sanger | A/C | A/C | C | A/C | C | A/C | C |
| Marker283629 | G/T | ddRAD-seq | G/T | G/T | T | G/T | T | G/T | G/T |
| | | Sanger | G/T | G/T | T | G/T | T | G/T | G/T |
| Marker19119 | G/T | ddRAD-seq | G/T | G/T | T | G/T | T | G/T | G/T |
| | | Sanger | G/T | G/T | T | G/T | T | G/T | G/T |
Inconsistent genotypes are marked with red color and the dash line indicates a missing genotype. Sequencing were conducted by Sangon Biotech (Shanghai) Co., Ltd.

## Slide 4
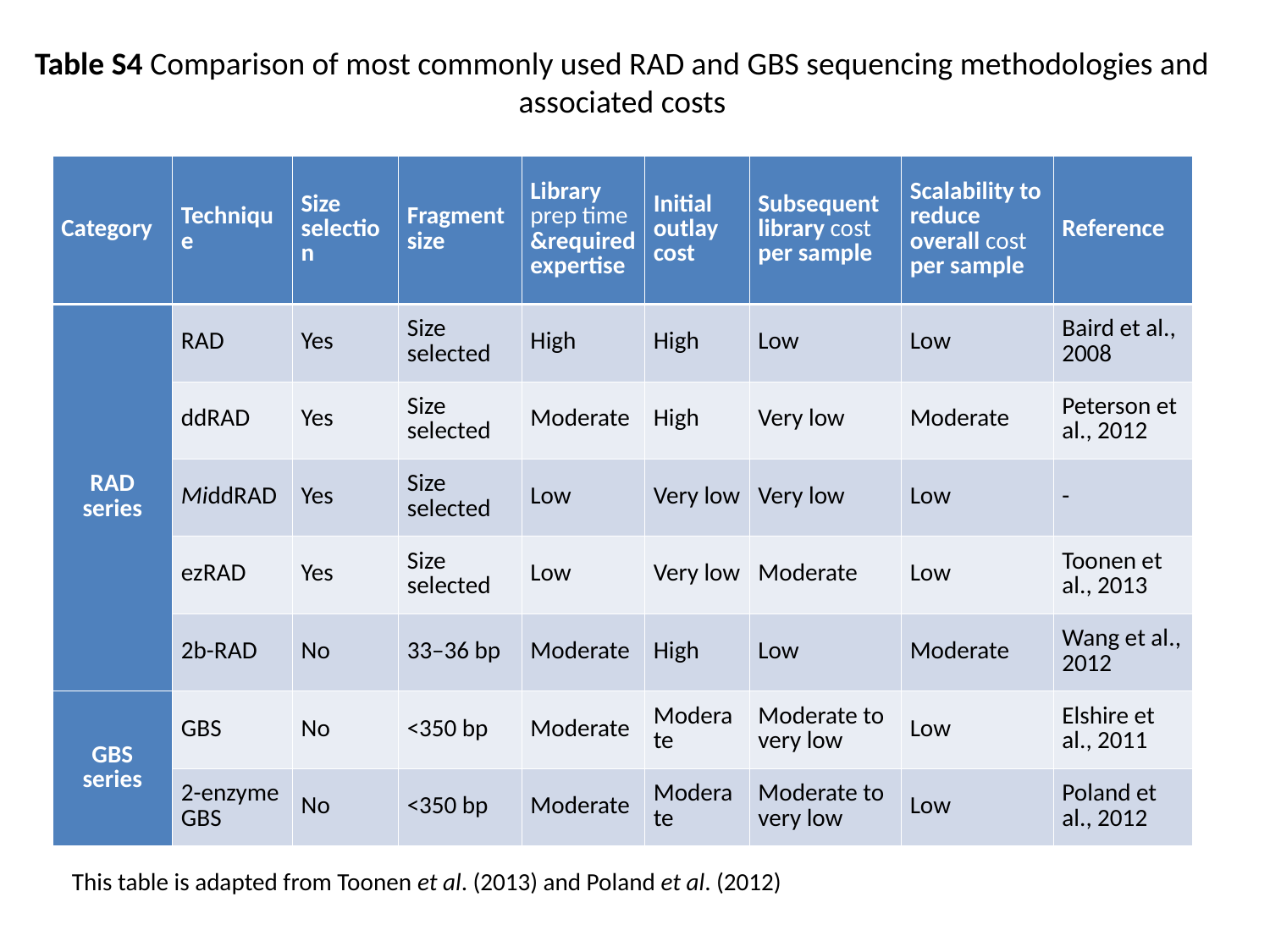

Table S4 Comparison of most commonly used RAD and GBS sequencing methodologies and associated costs
| Category | Technique | Size selection | Fragment size | Library prep time &required expertise | Initial outlay cost | Subsequent library cost per sample | Scalability to reduce overall cost per sample | Reference |
| --- | --- | --- | --- | --- | --- | --- | --- | --- |
| RAD series | RAD | Yes | Size selected | High | High | Low | Low | Baird et al., 2008 |
| | ddRAD | Yes | Size selected | Moderate | High | Very low | Moderate | Peterson et al., 2012 |
| | MiddRAD | Yes | Size selected | Low | Very low | Very low | Low | - |
| | ezRAD | Yes | Size selected | Low | Very low | Moderate | Low | Toonen et al., 2013 |
| | 2b-RAD | No | 33–36 bp | Moderate | High | Low | Moderate | Wang et al., 2012 |
| GBS series | GBS | No | <350 bp | Moderate | Moderate | Moderate to very low | Low | Elshire et al., 2011 |
| | 2-enzyme GBS | No | <350 bp | Moderate | Moderate | Moderate to very low | Low | Poland et al., 2012 |
This table is adapted from Toonen et al. (2013) and Poland et al. (2012)

## Slide 5
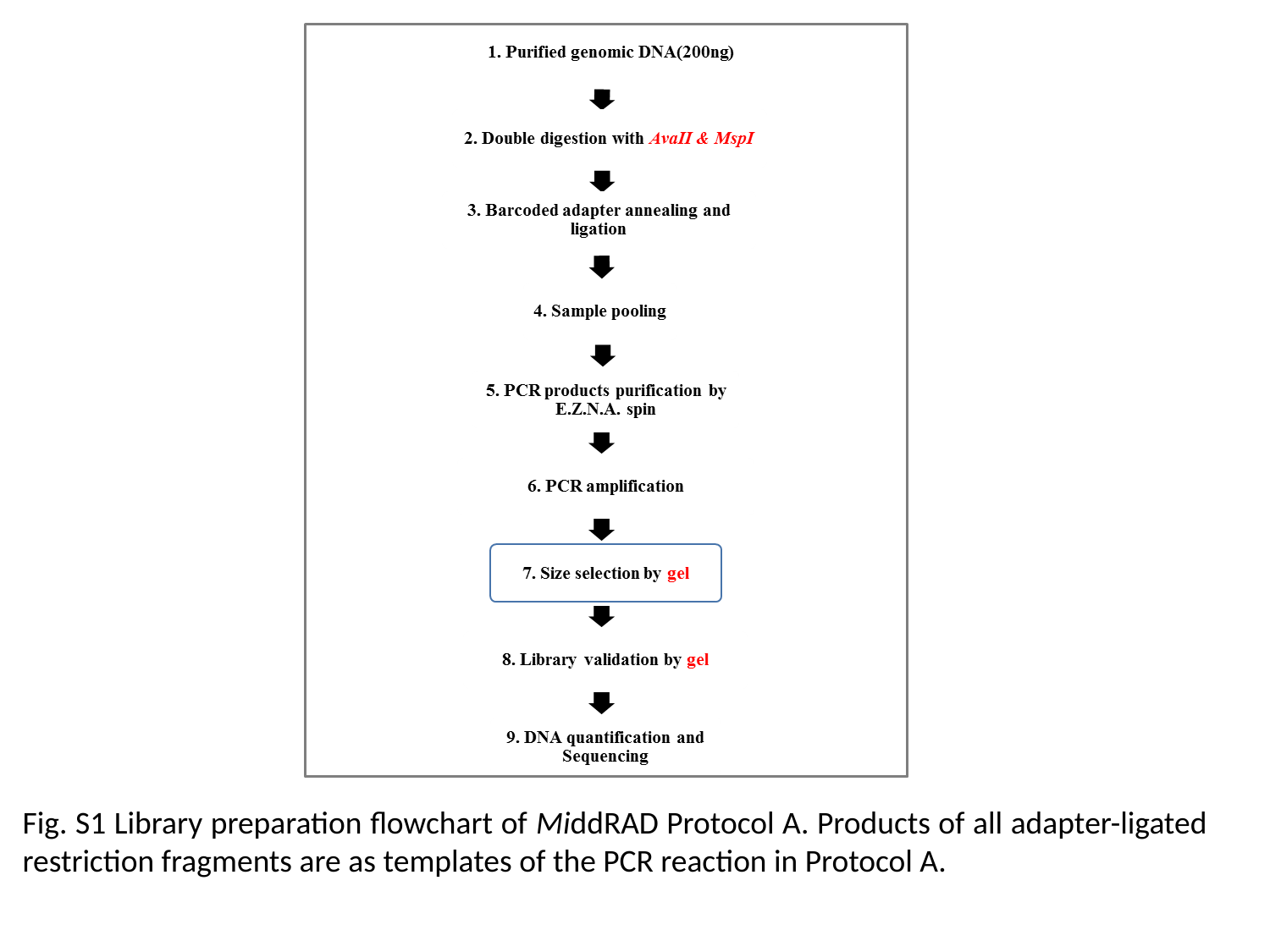

Fig. S1 Library preparation flowchart of MiddRAD Protocol A. Products of all adapter-ligated restriction fragments are as templates of the PCR reaction in Protocol A.

## Slide 6
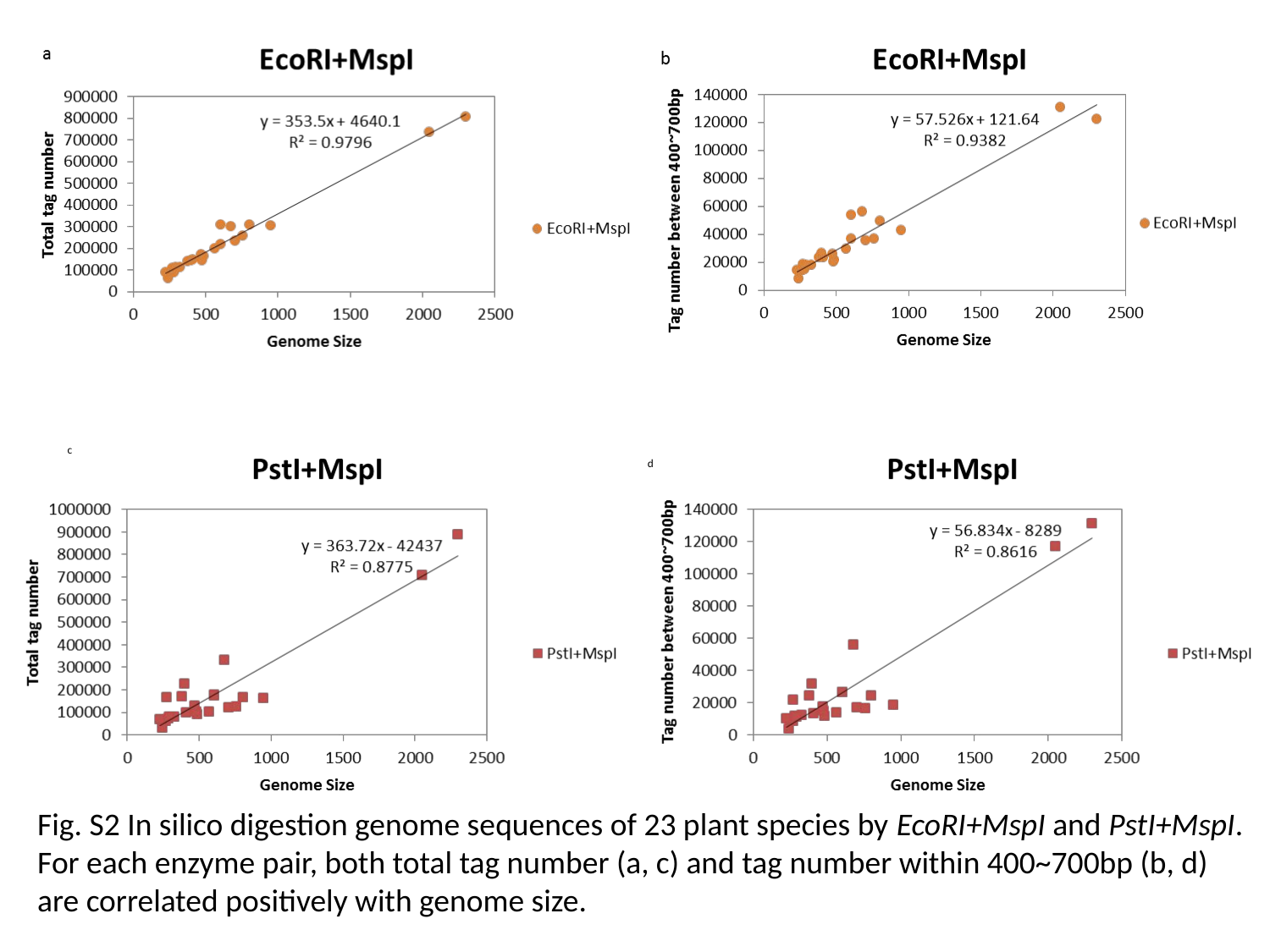

c
d
Fig. S2 In silico digestion genome sequences of 23 plant species by EcoRI+MspI and PstI+MspI. For each enzyme pair, both total tag number (a, c) and tag number within 400~700bp (b, d) are correlated positively with genome size.

## Slide 7
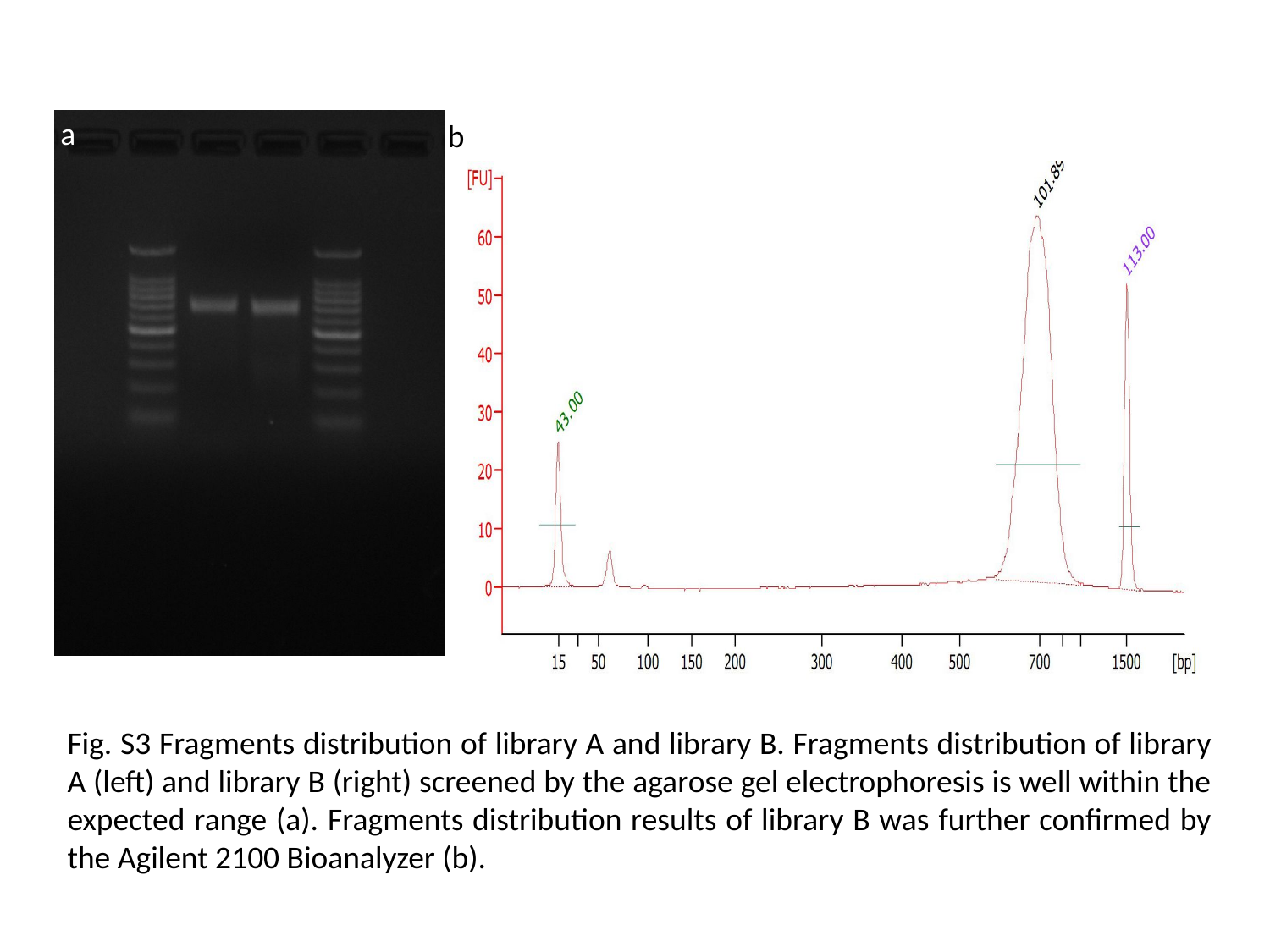

a
b
Fig. S3 Fragments distribution of library A and library B. Fragments distribution of library A (left) and library B (right) screened by the agarose gel electrophoresis is well within the expected range (a). Fragments distribution results of library B was further confirmed by the Agilent 2100 Bioanalyzer (b).

## Slide 8
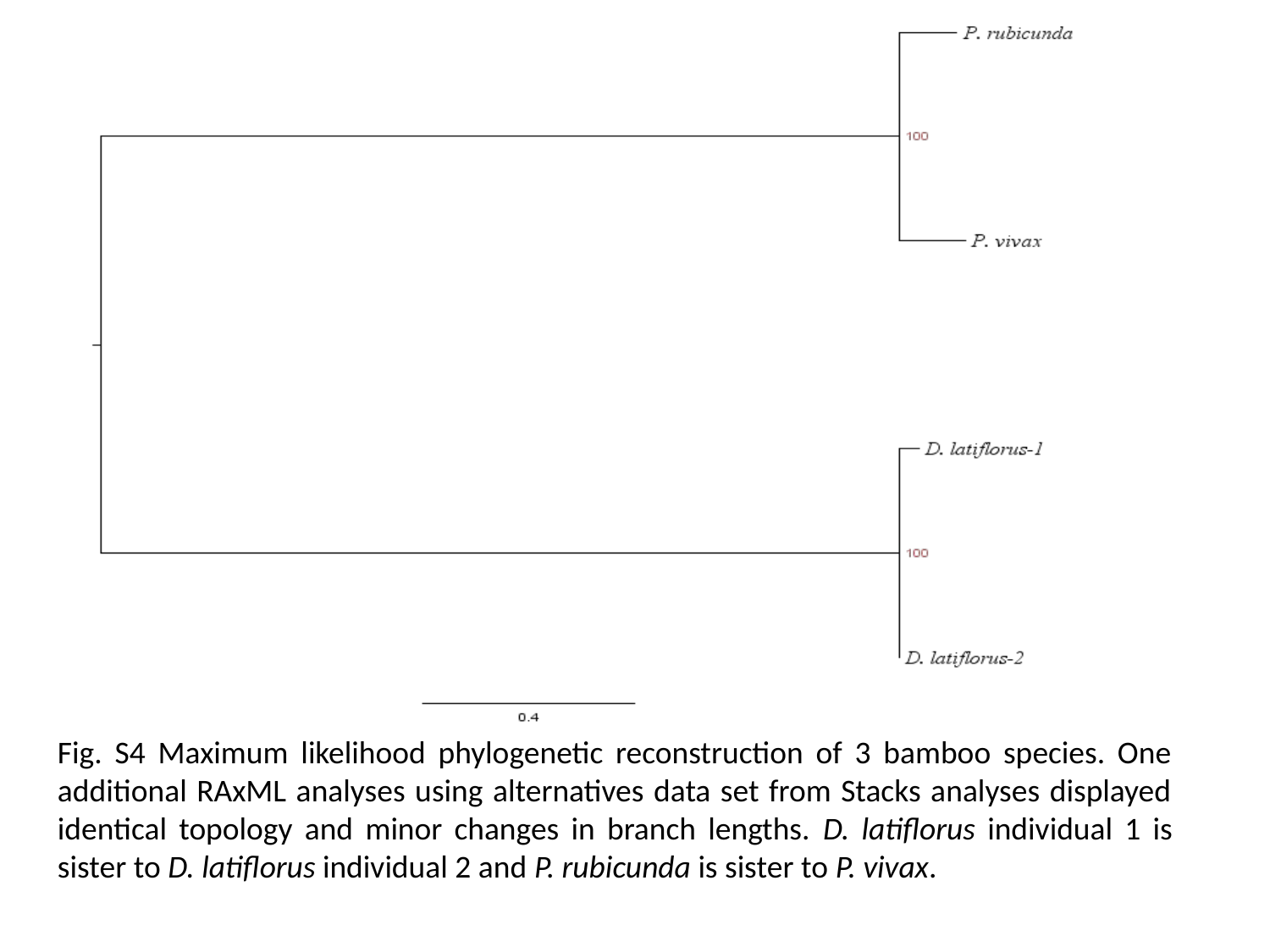

Fig. S4 Maximum likelihood phylogenetic reconstruction of 3 bamboo species. One additional RAxML analyses using alternatives data set from Stacks analyses displayed identical topology and minor changes in branch lengths. D. latiflorus individual 1 is sister to D. latiflorus individual 2 and P. rubicunda is sister to P. vivax.
